# Pancreatic ductal deletion of S100A9 alleviates acute pancreatitis by targeting VNN1-mediated ROS release to inhibit NLRP3 activation

**DOI:** 10.7150/thno.54245

**Published:** 2021-03-04

**Authors:** Hong Xiang, Fangyue Guo, Xufeng Tao, Qi Zhou, Shilin Xia, Dawei Deng, Lunxu Li, Dong Shang

**Affiliations:** 1 Laboratory of Integrative Medicine, First Affiliated Hospital of Dalian Medical University, Dalian, 116011, China.; 2 Institute (College) of Integrative Medicine, Dalian Medical University, Dalian, 116044, China.; 3 School of Chemical Engineering, Dalian University of Technology, Dalian, 116024, China.; 4 Department of General Surgery, First Affiliated Hospital of Dalian Medical University, Dalian, 116011, China.

**Keywords:** acute pancreatitis, acinar cells, duct cells, S100A9, VNN1

## Abstract

Recent studies have proven that the overall pathophysiology of pancreatitis involves not only the pancreatic acinar cells but also duct cells, however, pancreatic duct contribution in acinar cells homeostasis is poorly known and the molecular mechanisms leading to acinar insult and acute pancreatitis (AP) are unclear. Our previous work also showed that S100A9 protein level was notably increased in AP rat pancreas through iTRAQ-based quantitative proteomic analysis. Therefore, we investigated the actions of injured duct cells on acinar cells and the S100A9-related effects and mechanisms underlying AP pathology in the present paper.

**Methods:** In this study, we constructed S100A9 knockout (s100a9^-/-^) mice and an *in vitro* coculture system for pancreatic duct cells and acinar cells. Moreover, a variety of small molecular inhibitors of S100A9 were screened from ChemDiv through molecular docking and virtual screening methods.

**Results:** We found that the upregulation of S100A9 induces cell injury and inflammatory response via NLRP3 activation by targeting VNN1-mediated ROS release; and loss of S100A9 decreases AP injury* in vitro* and *in vivo*. Moreover, molecular docking and mutant plasmid experiments proved that S100A9 has a direct interaction with VNN1 through the salt bridges formation of Lys57 and Glu92 residues in S100A9 protein. We further found that compounds C_42_H_60_N_4_O_6_ and C_28_H_29_F_3_N_4_O_5_S can significantly improve AP injury *in vitro* and *in vivo* through inhibiting S100A9-VNN1 interaction.

**Conclusions:** Our study showed the important regulatory effect of S100A9 on pancreatic duct injury during AP and revealed that inhibition of the S100A9-VNN1 interaction may be a key therapeutic target for this disease.

## Introduction

Acute pancreatitis (AP) is a disorder that may cause local or systemic inflammatory response syndrome (SIRS) 1,2. Currently, AP is the second leading cause of total hospitalizations, the fifth leading cause of in-hospital deaths and the largest contributor to hospital costs, representing a huge burden on healthcare services around the world 3. Most AP patients recover within a week, whereas approximately 20% of AP patients will progress to severe acute pancreatitis (SAP), with a mortality risk as high as 30% 1,4. Patients with SAP have a high risk of multiorgan failure and need invasive interventions for local and systemic complications 5. Although our understanding of SAP pathogenesis has gradually improved in recent years, there is not yet a generally recognized specific therapy for this disease 6,7. Therefore, inhibition of AP exacerbation through early intervention is a promising treatment for SAP.

Acinar cells that secrete enzymes and the system of epithelial ductal cells that secrete fluid carrying digestive enzymes in the gut form the exocrine pancreas, which is the major target of AP 8-10. Most studies focus on the function and injury of acinar cells in AP; however, increasing evidence shows that the pancreatic duct is the primary target of stressors, which may play a key role in the exacerbation of AP 8,11-13. Previous studies have shown that the main function of the pancreatic duct is fluid and HCO3^-^ secretion, which is essential for normal digestion, and normal ductal secretion is a crucial factor for protecting the pancreas from AP 13-15. However, there are still few papers reporting the effects of digestive enzymes on the pancreatic duct in AP and the effects of this damage on acinar cells.

Our previous work showed through iTRAQ-based quantitative proteomic analysis that the level of S100 calcium binding protein A9 (S100A9) is significantly upregulated in the pancreas in rats with AP 16. S100A9, also named myeloid-related protein 14 (MRP14), is a Ca^2+^-binding protein belonging to the S100 family, and it plays an important role in the development of inflammation and immune responses 17-19. In pancreatic diseases, previous studies have indicated that pancreatic ductal adenocarcinoma (PDAC) patients with elevated levels of S100A9 in the ductal fluid have a notably worse prognosis 20 and the plasma level of S100A9 is also significantly increased in AP mice and patients 21,22. However, there is still no relevant research on the expression levels and effects of S100A9 in injured pancreatic duct cells during AP.

Therefore, in the present paper, we constructed S100A9 knockout (s100a9^-/-^) mice and an* in vitro* coculture system for pancreatic duct cells and acinar cells to study the effects of injured duct cells on acinar cells and the S100A9-related effects and mechanisms underlying AP pathology. In addition, small molecular compounds including C_42_H_60_N_4_O_6_ and C_28_H_29_F_3_N_4_O_5_S based on the mechanisms of S100A9 in AP were screened and verified, which provides an experimental basis for clinical treatment of AP in the future.

## Materials and Methods

### Cell culture

H6C7 (ATCC; VA, USA), a human pancreatic duct cell line, was cultured in DMEM with 10% fetal bovine serum. Primary acinar cells were extracted and cultured in Waymouth's medium with 2.5% fetal bovine serum according to our previous method 23. Briefly, the mouse pancreas was digested with collagenase solution containing 200 U/mL collagenase IA (Sigma; CA, USA), and 0.25 mg/mL soybean trypsin inhibitor (Gibco; CA, USA) at 37°C for 20 min. The disrupted tissue was then filtered through 70 μm nylon meshes (BD; NJ, USA), and the cell suspension was centrifuged at 450 g for 2 min. Next, the cell pellet was resuspended in Waymouth's medium and seeded in a culture plate. After 24 h of culture, acinar cells in medium were reseeded in a new plate that was precoated with type I collagen (Solarbio; Beijing, China) (50 μg/mL) for another 24 h of culture. After 1 days of culture, primary acinar cells were used for other experiments.

### Sodium taurocholate (STC)-induced cell injury

The IC50 value of STC in H6C7 cells was detected using the MTT method. Briefly, H6C7 cells were seeded in 96-well plates for 24 h at a density of 1 × 10^5^/mL. STC (Solarbio; Beijing, China) was added at gradually increasing concentrations (0, 200, 400, 600, 800, 1,000, 1,200, 1,400, 1,600, 1,800 and 2,000 μM) for 1 h of treatment. Next, 10 μL of 5 mg/mL MTT solution was added to each well for 4 h in a 37 °C incubator. Subsequently, DMSO was added to dissolve the formazan crystals, and the OD value was measured with a microplate reader (BioTek; VT, USA) at 490 nm. Finally, we used the IC50 value of STC to establish the *in vitro* injury model. Cell morphology was observed and imaged using a phase contrast microscope (Olympus; Tokyo, Japan).

### Ca^2+^, apoptosis and ROS assays

Intracellular Ca^2+^ detection was carried out by using Fluo-3 AM reagent (KeyGEN; Nanjing, China). H6C7 cells were collected, loaded for 45 min at 37°C with 5 μM Fluo-3 AM in culture medium supplemented with DMSO at 5 μM and Pluronic F-127 at 0.02%, washed with the same medium, and allowed to equilibrate for 30 min. The fluorescence was measured at an excitation wavelength of 490 nm and an emission wavelength of 515 nm using a confocal microscope with laser scanning. Cell apoptosis was detected by using an Annexin V-FITC/PI apoptosis detection kit (Meilun; Dalian, China). H6C7 cells were collected and then stained with Annexin V-FITC (5 µL) and PI (5 µL). After 15 min of incubation in a dark box at room temperature, the stained cells were analyzed by flow cytometry (BD; NJ, USA). In addition, ROS release was assayed by an ROS assay kit (AmyJet; Wuhan, China). H6C7 cells in different groups were collected and then incubated in culture medium with 1× ROS Label for 30 min at 37 °C. ROS levels in the treated cells were finally analyzed using flow cytometry (BD; NJ, USA) or fluorescence microscopy (Olympus; Tokyo, Japan).

### Immunofluorescence (IF) staining

H6C7 cells were incubated with anti-rabbit amylase (AMS) and anti-mouse CK19 antibodies (Abcam; Cambridge, UK) in a moist box overnight at 4 °C and then treated with TRITC-conjugated goat anti-rabbit IgG and FITC-conjugated goat anti-mouse IgG for 1 h at 37 °C. The samples were then restained with DAPI (5 μg/mL) for 5 min and observed under an Olympus BX63 fluorescence microscope (Olympus; Tokyo, Japan).

### Enzyme linked immunosorbent assay (ELISA)

The contents of S100A8, S100A9 and S100A8/S100A9 Heterodimer (S100A8/9 dimer) in H6C7 cells supernatant were assayed by using according ELISA kits (R&D Systems, MN, USA). Moreover, vanin1 (VNN1) level in H6C7 cells was also detected by using a Human VNN1 ELISA kit (Jianglailab, Shanghai, China).

### Plasmid transfection experiment

To identify the gene functions of S100A8, S100A9 and VNN1, H6C7 cells were seeded in 6-well plates for 24 h and then transfected for gene silencing and overexpression. The transfected plasmids containing shRNA-*S100A8* (sh*S100A8*), shRNA-*S100A9* (sh*S100A9*) or shRNA-*VNN1* (sh*VNN1*) were used for gene silencing, and those containing S100A8-DNA (*S100A8*) or S100A9-DNA (*S100A9*) were used for gene overexpression. H6C7 cells transfected with empty vector were used as a negative control (NC). These plasmids were purchased from GenePharma (Shanghai, China). After 24 h of transfection, the cells were subjected to the other treatments used in this study.

### IP and LC-MS/MS analysis

After transfection with the S100A9 overexpression plasmid (*S100A9*) for 24 h, H6C7 cells were harvested and lysed. Anti-S100A9 or IgG antibodies (Abcam; Cambridge, UK) were added to the lysis solution for antibody immobilization at 4 °C overnight. After incubation with Protein A/G Magnetic Beads (MedChemExpress; Shanghai, China) at 4 °C for 3 h, the protein complex was centrifuged and then washed 3 times with Pierce IP Lysis Buffer (Thermo; MA, USA) for SDS-PAGE analysis. Finally, the SDS-PAGE gel was subjected to silver staining to detect the difference in protein binding between the S100A9 and IgG antibodies. In addition, after pull-down experiments, the two protein samples underwent reductive alkylation and enzymolysis. Moreover, to detect the polypeptide sequence of protein samples, LC-MS/MS analysis was implemented. The polypeptide sequence was identified using ProteinPilot software of the AB SCIEX Triple TOF™ 5600 plus MS system (MA, USA).

### Determination of cysteamine level

Cysteamine level in H6C7 cells was detected through High-Performance Liquid Chromatography (HPLC) according to previous methods 24,25.

### Co-IP assay

The lysates of H6C7 cells were centrifuged at 12,000 rpm for 10 min, and the supernatant was subsequently collected. S100A9 or VNN1 antibody (10 μg) (Proteintech; Wuhan, China) was then added to the remaining lysate for overnight incubation at 4 °C after a small amount of lysate was collected for other experiments. Protein A/G Magnetic Beads (MedChemExpress; Shanghai, China) were washed repeatedly by PBS with 0.5% triton, added to the cell lysate and incubated at room temperature for 2 hours. Supernatant and magnetic beads were then separated on a magnetic frame, and the beads were washed three times with 1 mL of Pierce IP Lysis Buffer (Thermo; MA, USA). Then, 100 μL of 2× SDS loading buffer was added, and samples were incubated at 95 °C for 5 min and subjected to western blotting analysis.

### Quantitative real-time PCR analysis

Total RNA was isolated using RNAex Pro RNA reagent (Accurate Biology; Hunan, China) according to the manufacturer's instructions. RNA was then reverse-transcribed using the PrimeScript^®^ RT reagent kit (TaKaRa, Dalian, China), and mRNA expression levels of different genes were quantified using SYBR^®^ Premix Ex Taq™ II (Tli RNaseH Plus) (TaKaRa, Dalian, China) in an ABI 7500 Real-Time PCR System (Applied Biosystems; CA, USA). The primer sequences for the genes are listed in Supplementary Table S1, and they were purchased from Sangon Biotech (Shanghai, China). All gene expression levels were normalized to β-actin, and the fold changes between the different groups were calculated using a standard curve for quantitative analysis.

### Glutathione (GSH) and γ-glutamylcysteine synthetase (γ-GCS) assays

GSH and γ-GCS assays were detected by using a micro reduced GSH assay kit (Solarbio; Beijing, China) and γ-GCS assay kit (Jiancheng; Nanjing, China), respectively, according to the manufacturer's instructions. Finally, GSH and γ-GCS levels were measured at wavelengths of 636 nm and 412 nm, respectively, with a microplate reader (BioTek; VT, USA).

### Western blotting

Protein samples were extracted, and then separated by SDS-PAGE and transferred onto PVDF membranes (Millipore; MA, USA). After blocking with 5% skim milk, the membranes were incubated with primary antibodies (1: 1,000 dilution in TBST) at 4 °C overnight. Next, the membranes were incubated with secondary antibody for 2 h at room temperature. Finally, protein expression on the membranes was visualized with enhanced ECL using an imaging system (Tanon 4200; Shanghai, China). β-actin was used as the internal control.

### Animals

Wild-type (WT) C57BL/6 and s100a9^-/-^ C57BL/6 mice (male, 28-35 g) were purchased from Cyagen Biosciences Inc. (Guangzhou, China). All mice were maintained under specific pathogen-free conditions at Cyagen Model Biological Research Center (Taicang) Co., Ltd. All experiments were performed in strict accordance with the People's Republic of China Legislation Regarding the Use and Care of Laboratory Animals. Mice were anesthetized by intraperitoneal administration of 1.25% trioxyethanol (0.2 mL/10 g). We constructed an AP mouse model according to a previous method 26. Briefly, the common hepatic duct was temporarily clamped at the liver hilum to prevent hepatic reflux after the anesthetized mice underwent a midline incision, and 10 μL of 5% STC (Solarbio; Beijing, China) or 0.9% sodium chloride was then infused into the pancreatic duct for 10 min. The catheter and the common hepatic duct clamp were removed after the injection. The mice were sacrificed after 48 h, and pancreas and serum were then collected.

To evaluate the effects of S100A9 in AP mice, α-amylase (α-AMS) and lipase (LPS) levels in the serum and inflammatory factor (IL-1β, IL-6, IL-8 and IL-18) contents in the pancreatic tissue were all detected with the corresponding kits. Moreover, we assayed oxidative stress and pancreatic injury through ROS, γ-GCS and GSH kits and HE staining, respectively. In addition, we used IF and immunohistochemistry (IHC) staining to detect the protein expression levels of S100A9, VNN1 and NLR family, pyrin domain containing 3 (NLRP3) according to the above methods.

### Molecular docking

The 3D structures of the VNN1 protein (PDB ID: 4CYF) and S100A9 protein (PDB ID: 5I8N) were downloaded from the RCSB Protein Data Bank. Protein-protein docking in the ClusPro server 27,28 was used for molecular docking simulations and predicting the binding affinity for the complex. For protein docking, the smaller protein (a smaller number of residues) is usually set as the ligand and the other protein is set as the receptor. The ligand was subjected to 70,000 rotations. For each rotation, the ligand was translated in the x, y, and z axes relative to the receptor on a grid. One translation with the best score was chosen from each rotation. Of the 70,000 rotations, the 1,000 rotation/translation combinations that had the lowest scores were chosen. Then, greedy clustering of these 1,000 ligand positions with a 9 Å C-alpha RMSD radius was performed to find the ligand positions with the most “neighbors” in 9 Å, i.e., cluster centers. The top ten cluster centers with most cluster members were then retrieved and inspected visually one by one. The intermolecular contacts from the most likely pose were further evaluated. The docked structures and interface residues were analyzed using MOE v2018.01 29, and molecular graphics were generated with PyMOL.

### Virtual screening

The dock module in MOE v2015.1001 30 was used for structure-based VS (SBVS). The structure of S100A9 was defined as a receptor, and 100,000 compounds from Chemdiv were used as the VS library. The binding site of the receptor was selected near the residues that were the interaction sites between S100A9 and VNN1. All compounds were prepared with the Wash module in MOE. After that, all compounds were first ranked by high-throughput rigid docking with London dG scoring, and then the top 10K compounds were selected for flexible docking with the “induced fit” protocol. Prior to docking, the force field of AMBER12: EHT and the implicit solvation model of the reaction field (R-field) were selected. The protonation state of the protein and the orientation of the hydrogens were optimized by the LigX module at a pH of 7 and a temperature of 300 K. For flexible docking, the docked poses were ranked by London dG scoring first, and then a force field refinement was carried out on the top 10 poses, followed by a rescoring of GBVI/WSA dG. The best ranked pose was selected as the final pose.

### Toxicology and pharmacodynamics of small molecule compounds

To evaluate the toxicology of small molecule compounds, C57BL/6 mice were divided into Control (ctrl) and Compound groups. Compounds were dissolved in 0.5% CMC-Na solution, and they were given orally to mice (10 mg/kg/day, 2 days). Serum, heart, liver, spleen, lung, kidney, brain, intestines and pancreas were collected after the mice were killing under anesthesia. α-AMS, LPS, ALT, AST, BUN and CRE levels in serums were assayed by using according kits, and the tissues were performed HE staining. In addition, to evaluate the pharmacodynamics of small molecule compounds, compounds were given orally to mice (10 mg/kg/day, 2 days) after the mice suffered from AP injury. Finally, Serum and pancreas were collected to detect α-AMS, LPS, γ-GCS, GSH, inflammatory factors and NLRP3 levels.

### Statistical analysis

Data were analyzed using GraphPad Prism 6.0 software (GraphPad; CA, USA) and expressed as the mean ± SEM. Differences among multiple groups were determined using one-way ANOVA, and differences between two groups were analyzed using an unpaired Student's t-test. P values of <0.05 or <0.01 were considered statistically significant.

## Results

### Loss of S100A9 decreases pancreatic injury in AP mice

**Figure 1:**
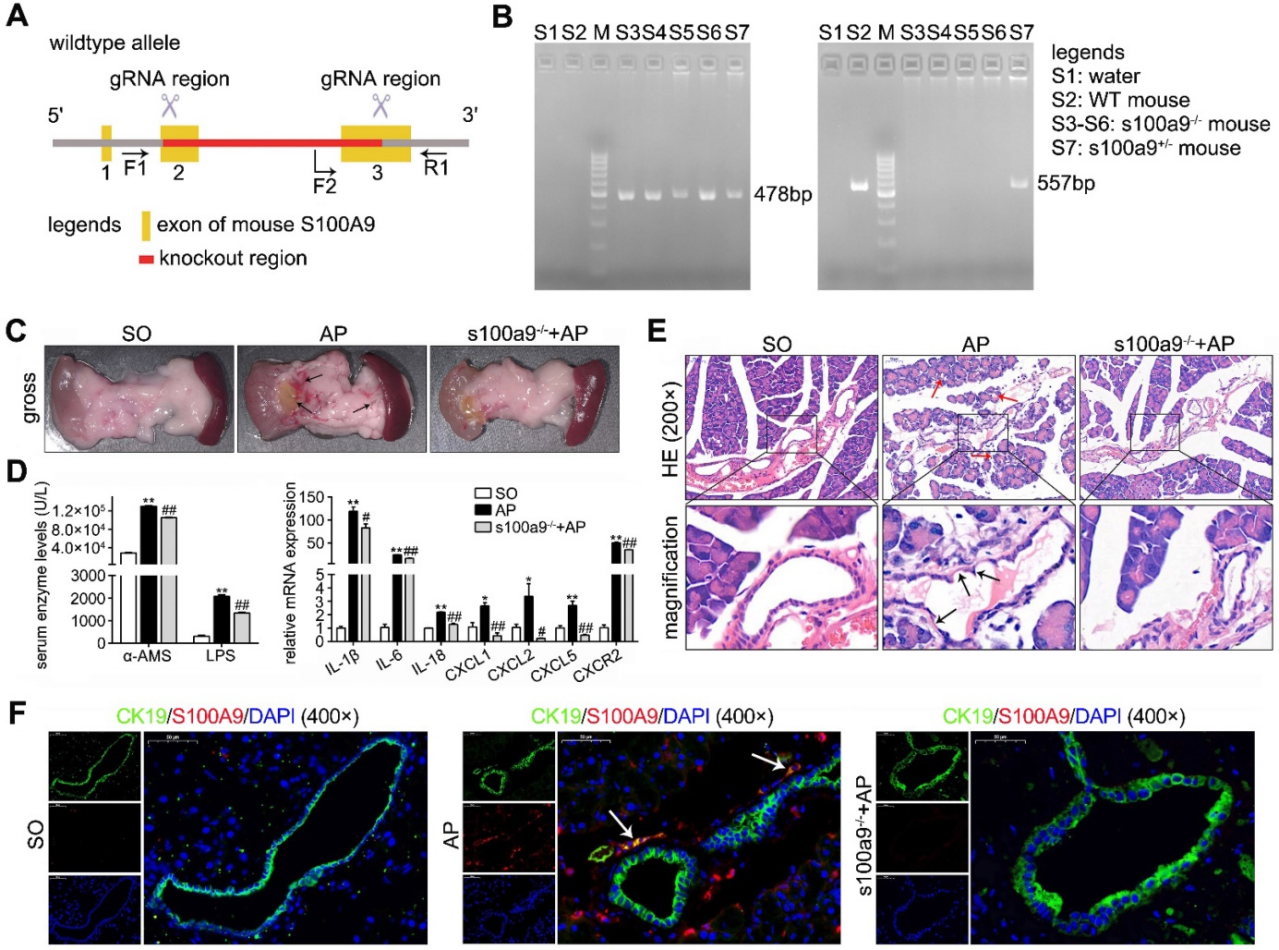
** Loss of S100A9 decreases pancreatic injury in AP mice.** (A) Overview of the targeting strategy for constructing s100a9^-/-^ mice. (B) PCR identification of homozygote, heterozygote and WT of s100a9^-/-^ mice. (C) Gross images of the pancreas indicated that the s100a9^-/-^ pancreas showed milder symptoms, including edema, hemorrhage and cholestasis. (D) s100a9^-/-^ mice showed lower serum enzymes (n = 8) and decreased inflammatory factor levels (n = 3). (E) HE staining images proved that both pancreatic duct and acinar injuries were obviously alleviated in the s100a9^-/-^ pancreas. (F) Double staining experiments showed that S100A9 protein levels were upregulated around the pancreatic duct (CK19-positive area). Data are presented as the mean ± SEM; *P < 0.05, **P < 0.01 *vs.* SO mice; ^#^P < 0.05, ^##^P < 0.01 *vs.* AP mice.

Given the importance of S100A9 in pancreatic duct injury, as shown in Figure 1A-B, we constructed S100A9 knockout mice (s100a9^-/-^ C57BL/6 mice). As shown in Figure 1C, pancreatic insults, including edema, hemorrhage and cholestasis, occurred in the AP mice (as shown by the arrow), but the s100a9^-/-^ pancreas showed milder symptoms than the AP pancreas. In addition, compared to those in AP mice, serum enzyme (α-AMS and LPS) levels and inflammatory factor (IL-1β, IL-6, IL-18, CXCL1, CXCL2, CXCL5 and CXCR2) levels were all significantly decreased in s100a9^-/-^ mice (Figure 1D). Interestingly, HE staining results showed obvious damage, such as pyknotic nuclei and concentrated cytoplasm in epithelial cells (as shown by the black arrow), in the pancreatic duct of AP mice, as well as apparent injuries in the acinar cells of AP mice, including vacuolization and increased neutrophils (as shown by the red arrow); however, these insults were obviously alleviated in s100a9^-/-^ mice (Figure 1E). Moreover, as shown in Figure 1F, the CK19/S100A9 double-staining experiment indicated that the S100A9 protein level was upregulated around the pancreatic duct (CK19-positive area) in AP mice, and some ductal cells expressed both CK19 and S100A9 (as shown by the arrow). These results prove that S100A9 upregulation aggravates pancreatic injury in AP mice, and the mechanism perhaps involving in the damage of ductal cells.

### Establishment of a coculture system for STC-injured ductal cells and primary acinar cells

**Figure 2:**
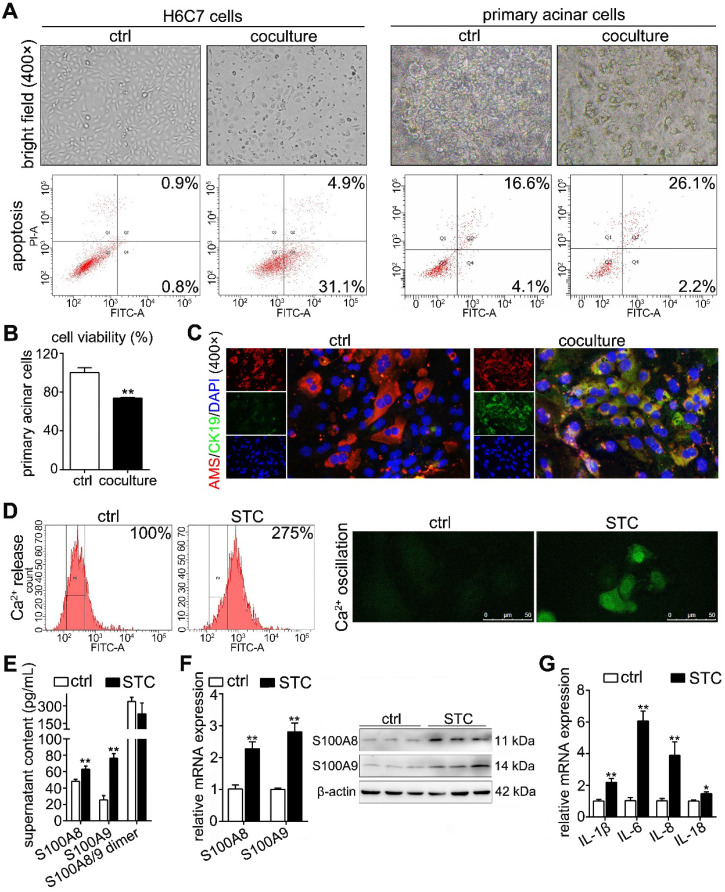
** Establishment of a coculture system for STC-injured ductal cells and primary acinar cells.** (A) Bright field images and flow cytometry results indicated that a large number of damaged and apoptotic cells occurred among H6C7 cells and primary acinar cells. (B) MTT results showed that the cell viability of primary acinar cells in the coculture system was significantly decreased compared with that of the ctrl group (n = 6). (C) AMS-CK19 double staining results showed that CK19 (green fluorescence, marker of ductal cells) was markedly upregulated in primary acinar cells. (D) Flow cytometry and laser confocal microscopy results showed that Ca^2+^ release and oscillation were all increased in STC-injured H6C7 cells. (E) ELISA results showed that single S100A8 and S100A9 levels were all significantly increased, but the S100A8/9 dimer level has no obvious change in STC-injured H6C7 cells (n = 5). (F) S100A8 and S100A9 mRNA and protein expression levels were notably increased in STC-injured H6C7 cells (n = 3). (G) Levels of inflammatory factors were markedly elevated in STC-injured H6C7 cells (n = 3). Data are presented as the mean ± SEM; *P < 0.05 and **P < 0.01 *vs.* ctrl group.

To observe the insult of digestive enzymes on the pancreatic duct in AP and the effects of this damage on acinar cells, we used STC to pretreat H6C7 cells and further constructed an* in vitro* coculture system for STC-injured H6C7 cells and primary acinar cells. As shown in Supplementary Figure S1A, STC-injured H6C7 cells and primary acinar cells were seeded into the lower chamber and upper chamber of the transwell plate, respectively. In this model, we used gradually increasing concentrations of STC to pretreat the H6C7 cells for 1 h and found that its IC50 value was 1,016 μM (Supplementary Figure S1B), which was chosen as the insult dose for H6C7 cells in follow-up experiments. After 3 days of coculture, bright field images (400× magnification) captured with an inverted microscope showed an obviously decreased number of normal cells and increased cell debris in H6C7 cells and massive cell atrophy and injury in acinar cells (Figure 2A). Flow cytometry results also indicated that there were a large number of apoptotic cells among H6C7 cells and acinar cells (Figure 2A). Moreover, the injured H6C7 cells caused a significant decrease in acinar cell viability (Figure 2B). In addition, as shown in Supplementary Figure S1C, IF staining proved that the expression of the ductal cell marker CK19 clearly decreased in STC-treated H6C7 cells. In addition, AMS-CK19 double staining results showed that CK19 (green fluorescence) was markedly upregulated in primary acinar cells (Figure 2C), which indicated that STC-injured H6C7 cells cause the development of acinar-to-ductal metaplasia (ADM) in primary acinar cells *in vitro*. S100A8 and S100A9 are Ca^2+^-binding proteins belonging to the S100 family; therefore, we assayed Ca^2+^ level and found that STC markedly increased Ca^2+^ release and oscillation (Figure 2D). We also detected the contents of S100A8, S100A9 and S100A8/9 dimer in supernatant of H6C7 cells, and found that single S100A8 and S100A9 levels were all significantly increased, but the S100A8/9 dimer level has no obvious change in STC-injured H6C7 cells compared with ctrl group (Figure 2E). Furthermore, we assayed the mRNA and protein expression levels of S100A8 and S100A9, and found that they were all significantly upregulated in STC-injured H6C7 cells (Figure 2F). We also detected inflammation in STC-injured H6C7 cells and found that the release of inflammatory factors, including IL-1β, IL-6, IL-8 and IL-18, was significantly elevated (Figure 2G). Therefore, STC damages H6C7 cells, possibly by increasing inflammatory response via upregulation of S100A8 and S100A9 expression levels.

### S100A8 and S100A9 are important elements in STC-induced ductal cell injury

**Figure 3:**
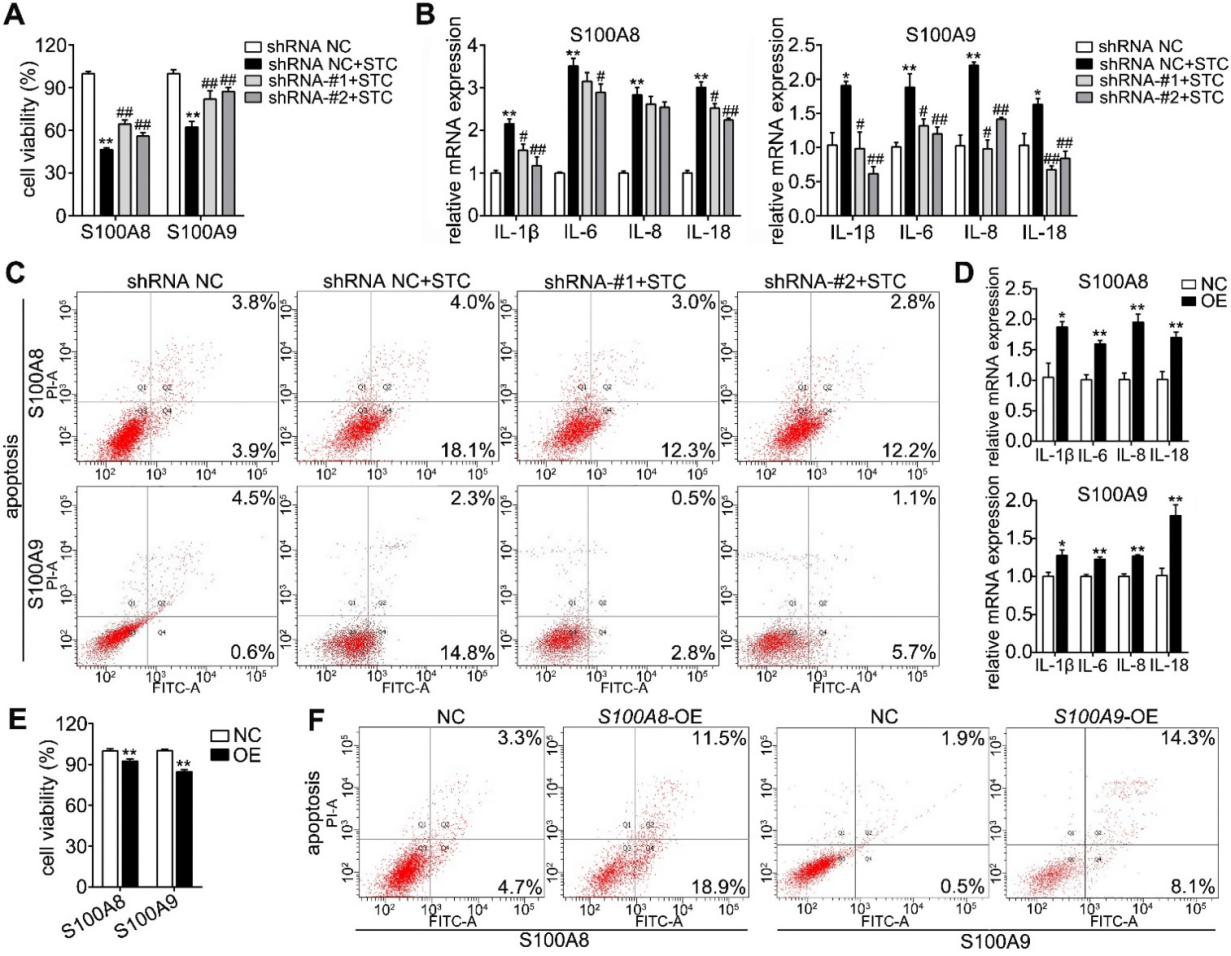
** S100A8 and S100A9 are important elements in STC-induced ductal cell injury.** (A) Knockdown of S100A8 or S100A9 significantly increased the viability of H6C7 cells after STC pretreatment (n = 6). (B) Knockdown of S100A8 or S100A9 markedly inhibited inflammatory factor releases (IL-1β, IL-6, IL-8 and IL-18) in STC-pretreated H6C7 cells (n = 3). (C) Knockdown of S100A8 or S100A9 notably decreased apoptosis in STC-pretreated H6C7 cells. (D) Inflammatory factors (IL-1β, IL-6, IL-8 and IL-18) levels were significantly elevated in S100A8 or S100A9-overexpressing H6C7 cells (n = 3). (E) Cell viability of H6C7 cells were notably decreased in S100A8 or S100A9-overexpressing H6C7 cells (n = 6). (F) Apoptosis in H6C7 cells was increased in S100A8 or S100A9-overexpressing H6C7 cells. Data are presented as the mean ± SEM, *P < 0.05 and **P < 0.01 *vs.* shRNA NC or NC group; ^#^P < 0.05 and^ ##^P < 0.01 *vs.* shRNA NC+STC group.

To further discuss the roles of S100A8 and S100A9 in STC-induced ductal cell injury, we knocked down and overexpressed their protein levels by transfecting shRNA and overexpression plasmids, respectively. First, we constructed knockdown plasmids (sh*S100A8* and sh*S100A9*) and overexpression plasmids (*S100A8* and *S100A9*). As shown in Supplementary Figure S2A-B, sh*S100A8*-#1, -#2 and sh*S100A9-#1,* -#2 significantly inhibited S100A8 and S100A9 expressions, respectively; and our results showed that knockdown of S100A8 or S100A9 significantly decreased STC-induced ductal cell injury and thereby increased cell viabilities (Figure 3A). Moreover, compared to STC-treated H6C7 cells, the cells in both the sh*S100A8* and sh*S100A9* groups markedly inhibited inflammatory factors release (IL-1β, IL-6 and IL-18) (Figure 3B). Meanwhile, sh*S100A9* also decreased IL-8 mRNA expression, but sh*S100A8* had no such effect (Figure 3B). Compared with STC group, cell apoptosis ratios were inhibited by both sh*S100A8* and sh*S100A9* plasmids (Figure 3C). In addition, S100A8 and S100A9 expressions were significantly upregulated in H6C7 cells after transfection with the *S100A8* and *S100A9* plasmid, respectively (Supplementary Figure S2C-D). Increased inflammatory factors release (Figure 3D) and decreased cell viabilities (Figure 3E) were all observed in overexpression plasmid transfected-H6C7 cells. Moreover, cell apoptosis ratios in overexpression groups were also upregulated compared to NC groups (Figure 3F). These results prove that S100A8 and S100A9 are important elements in STC-induced ductal cell injury, and inhibition of S100A8 or S100A9 expression can significantly improve ductal cell injury *in vitro*.

### STC promotes NLRP3 activation through increasing S100A9/VNN1 mediated ROS release

**Figure 4:**
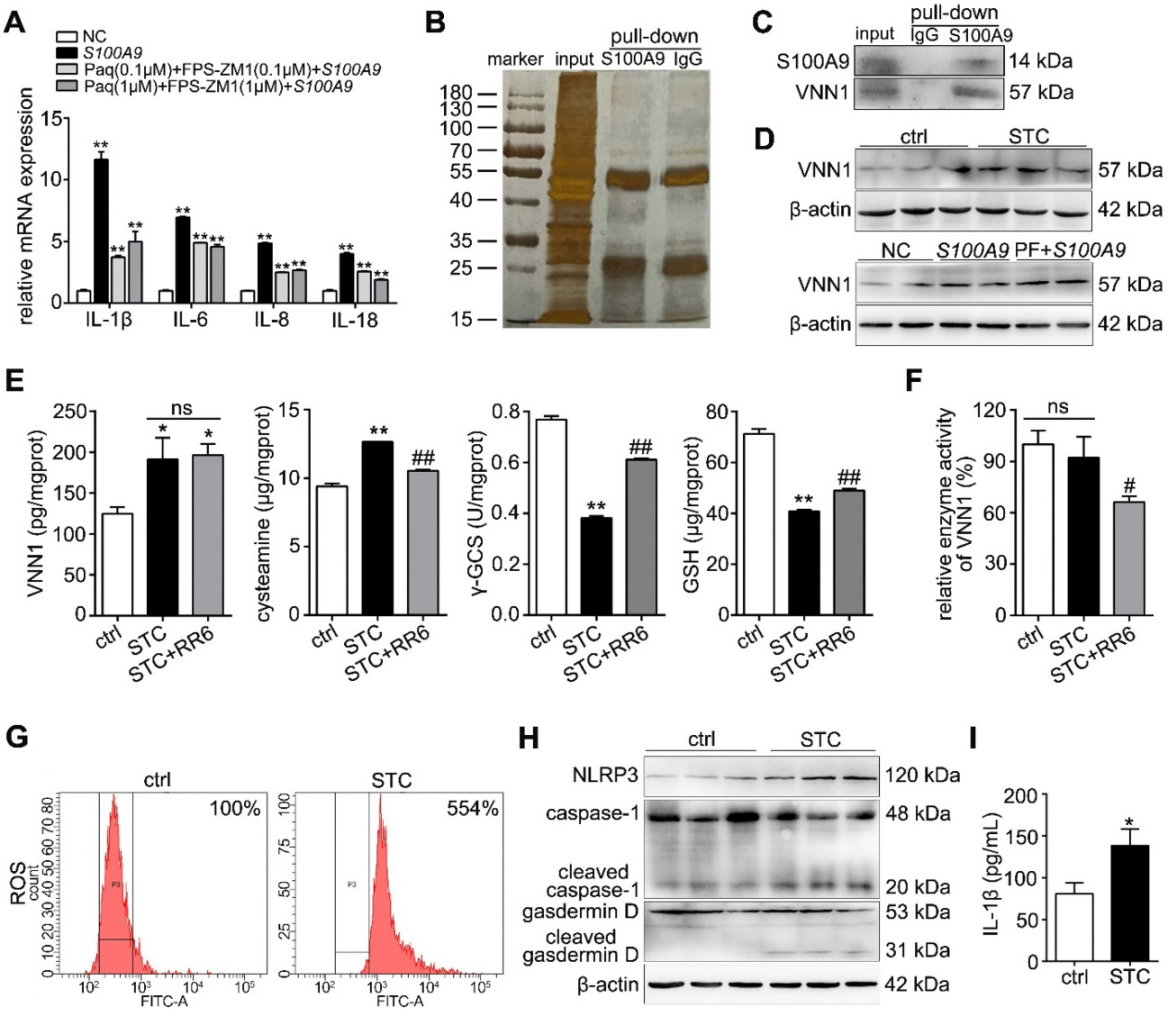
** STC promotes NLRP3 activation through increasing S100A9/ VNN1 mediated ROS release.** (A) Inflammatory factors (IL-1β, IL-6, IL-8 and IL-18) levels were remarkedly up-regulated in *S100A9* group after pre-treating with Paq and FPS-ZM1 compared to NC group (n = 3). (B) Silver staining results of S100A9 IP experiment. (C) Co-IP experiment results further indicated that the S100A9 protein can pull down the VNN1 protein. (D) VNN1 protein level was notably upregulated in STC-injured H6C7 cells, and its expression wasn't notably changed after inhibiting TLR4 and RAGE signalings. (E) STC induced the increases of VNN1 expression and cysteamine release, and the decreases of γ-GCS and GSH contents (n = 5). (F) RR6 inhibited VNN1 enzyme activity, but STC had on obvious effect on it (n = 5). (G-I) ROS release, and downstream NLRP3 expression, caspase-1 activation, gasdermin D cleavage and IL-1β level were all upregulated in STC-injured H6C7 cells (n = 5). Data are presented as the mean ± SEM; *P < 0.05 and **P < 0.01 *vs.* NC or ctrl group; ^#^P < 0.05 and^ ##^P < 0.01 *vs.* STC group.

S100A9 is known to be a ligand for toll-like receptor 4 (TLR4) and receptor of advanced glycation endproducts (RAGE), to stimulate pro-inflammatory response. We used paquinimod (Paq) and FPS-ZM1 to inhibit S100A9-mediated TLR4 and RAGE signaling pathways, and then detected inflammatory factor levels after overexpressing S100A9. As shown in Figure 4A, IL-1β, IL-6, IL-8 and IL-18 expressions were remarkedly decreased in Paq and FPS-ZM1 groups compared with S100A9-overexpressed group, but also significantly upregulated compared to NC group, which proved that there may exist other inflammatory signaling pathway independent of these two known receptors. In order to find the new pathway, we performed S100A9 IP experiment. As shown in Figure 4B, the silver staining results of the IP experiment indicated that S100A9 pulled down several different proteins compared to IgG. Thus, we further adopted LC-MS/MS to identify and analyze the binding proteins of S100A9 (Supplementary Figure S3A and Supplementary Table S2). As shown in Supplementary Figure S3B, we identified 56 and 20 kinds of proteins from IgG and S100A9 pull-down samples, respectively. Among them, 11 proteins, as shown in Supplementary Table S3, were unique proteins pulled down in the S100A9 group. After excluding 3 immunoglobulins (KVD39, IGK and IGHG3), we verified the mRNA expression levels of 8 other proteins through qPCR after transfection with the *S100A9* plasmid and found that HS71B, HNRH2, PABP1, TBB4A, TBA1C, TBD2A and VNN1 were all significantly upregulated, and the most variable gene was VNN1 (Supplementary Figure S3C-D). As shown in Figure 4C, the Co-IP results further indicated that the S100A9 protein can pull down the VNN1 protein, which verified the interaction between the S100A9 and VNN1 proteins. We also found that VNN1 protein level was notably upregulated in STC-injured H6C7 cells, and its expression wasn't notably changed after inhibiting TLR4 and RAGE signaling pathways (Figure 4D), which showed that S100A9/VNN1 signaling is another pathway that independent of TLR4 and RAGE. VNN1 can inhibit GSH and γ-GCS activities through generating cysteamine. Our results showed that STC induced the increases of VNN1 expression and cysteamine release, and the decreases of γ-GCS and GSH contents (Figure 4E). Meanwhile, RR6 didn't affect VNN1 content, but significantly inhibited cysteamine level and thereby increased γ-GCS and GSH releases in STC-injured H6C7 cells as an efficient VNN1 inhibitor (Figure 4E). Furthermore, as shown in Figure 4F, RR6 inhibited VNN1 enzyme activity, but STC didn't change it. In addition, we also found that ROS release (Figure 4G), and downstream NLRP3, gasdermin D cleavage, caspase-1 activation and IL-1β level (Figure 4H-I) were all upregulated in STC-injured H6C7 cells, which proved that STC promotes NLRP3 activation through increasing ROS release.

### Extracellular S100A9 increases apoptosis and inflammatory response by targeting VNN1

**Figure 5:**
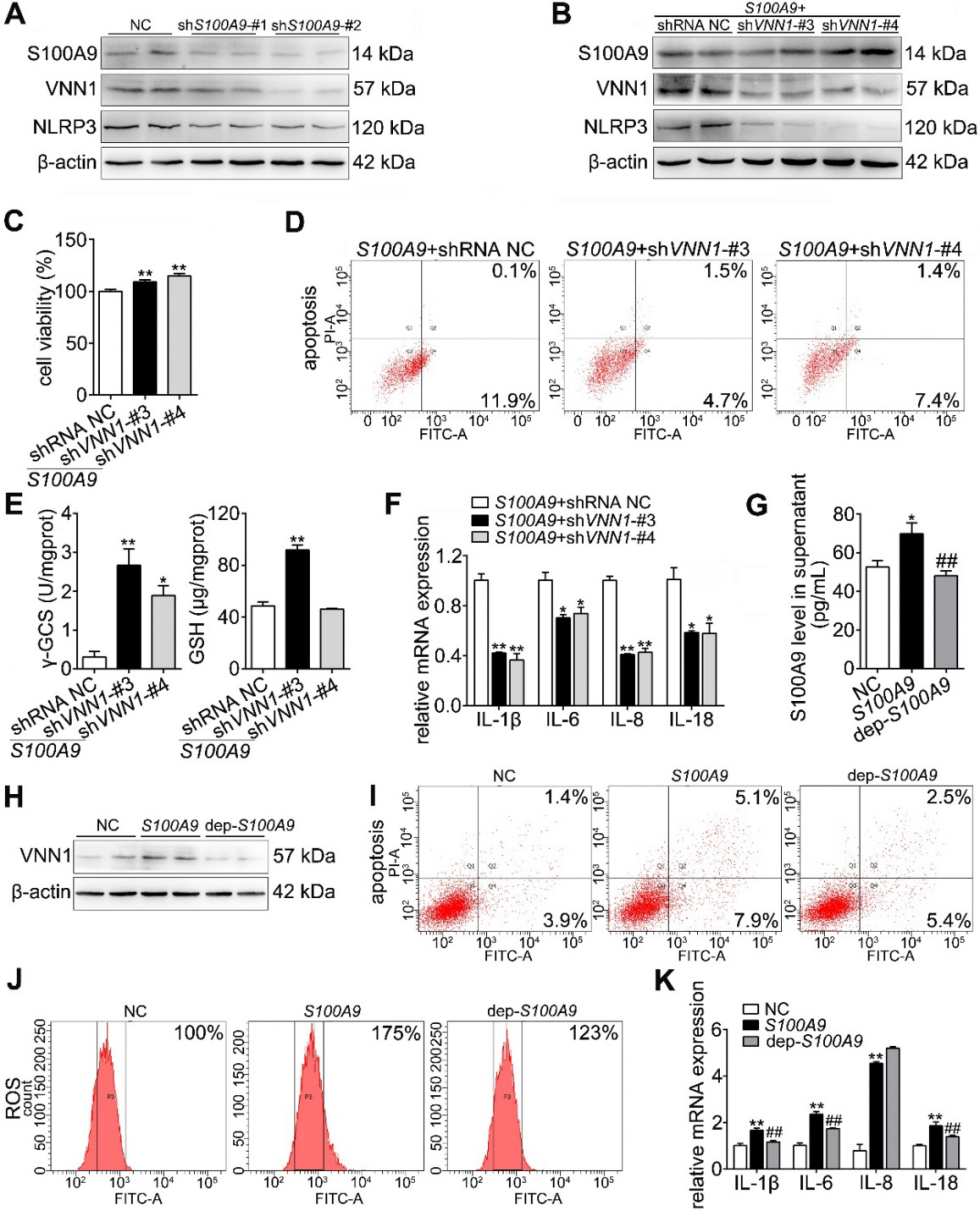
** Extracellular S100A9 increases apoptosis and inflammatory response by targeting VNN1.** (A) Protein expression levels of S100A9, VNN1 and NLRP3 were all downregulated after knocking down S100A9 in H6C7 cells. (B) Knockdown of VNN1 reduced VNN1 and NLRP3 protein levels in* S100A9* plasmid pretreated-H6C7 cells but had no obvious effects on S100A9 protein expression. (C-D) Knockdown of VNN1 significantly increased cell viability (n = 6), and decreased apoptosis in S100A9-overexpressing H6C7 cells. (E) Knockdown of VNN1 improved oxidative stress by upregulating γ-GCS and GSH release in S100A9-overexpressing H6C7 cells (n = 6). (F) Knockdown of VNN1 notably downregulated inflammatory factor levels in S100A9-overexpressing H6C7 cells (n = 3). (G) ELISA result showed that S100A9 release in dep-*S100A9* group is significantly downregulated compared with *S100A9* group (n = 5). (H-K) VNN1 expression, cell apoptosis, ROS and inflammatory factors (n = 3) were notably decreased in dep-*S100A9* group compared with *S100A9* group. Data are presented as the mean ± SEM; *P < 0.05 and **P < 0.01 *vs.* NC or *S100A9+*shRNA NC group; ^##^P < 0.01 *vs. S100A9* group.

As shown in Figure 5A, both sh*S100A9*-#1 and -#2 notably downregulated the protein expressions of S100A9, VNN1 and NLRP3. Furthermore, we constructed four knockdown plasmids (sh*VNN1*-#1, -#2, -#3 and -#4) and found that sh*VNN1*-#3 and -#4 had the strongest inhibitory effects on VNN1 mRNA (Supplementary Figure S4). To verify whether VNN1 is a downstream interacting protein of S100A9, we knocked down VNN1 expression in *S100A9* plasmid-pretreated H6C7 cells by transfection with sh*VNN1*-#3 and -#4 and then detected H6C7 cell viability and apoptosis. As shown in Figure 5B, western blotting results showed that sh*VNN1*s can inhibit VNN1 and NLRP3 protein levels in* S100A9* plasmid pretreated-H6C7 cells but has no obvious effect on S100A9 protein expression. Moreover, sh*VNN1*s significantly increased cell viability (Figure 5C) and decreased cell apoptosis (Figure 5D), which indicated that knockdown of VNN1 can inhibit S100A9 overexpression induced cell injury. In addition, knockdown of VNN1 notably improved oxidative stress by upregulating GSH and γ-GCS release (Figure 5E) and downregulated the levels of inflammatory factors, including IL-1β, IL-6, IL-8 and IL-18 (Figure 5F). These results further indicated that VNN1 is a downstream interacting protein of S100A9. In addition, S100A9 is a secretory protein, and it can be secreted out of the cells. In order to judge which part of S100A9 works with VNN1, we constructed a S100A9 plasmid of de-polypeptide (10 amino acids were removed after the start codon) (dep-*S100A9*), whose S100A9 protein secretion outside of the cell may decreased. As shown in Figure 5G, ELISA result showed that S100A9 protein secretion in cell supernatant of dep-*S100A9* group is significantly down-regulated compared with *S100A9* group. In addition, VNN1 expression, cell apoptosis, ROS and inflammatory factors are notably decreased in dep-*S100A9* group compared with *S100A9* group (Figure 5H-K). Importantly, considering that VNN1 is a GPI-anchored protein, these results proved that S100A9 exerts its actions mainly through the extracellular S100A9.

### Loss of S100A9 inhibits NLRP3 activation by decreasing VNN1-mediated ROS release

**Figure 6:**
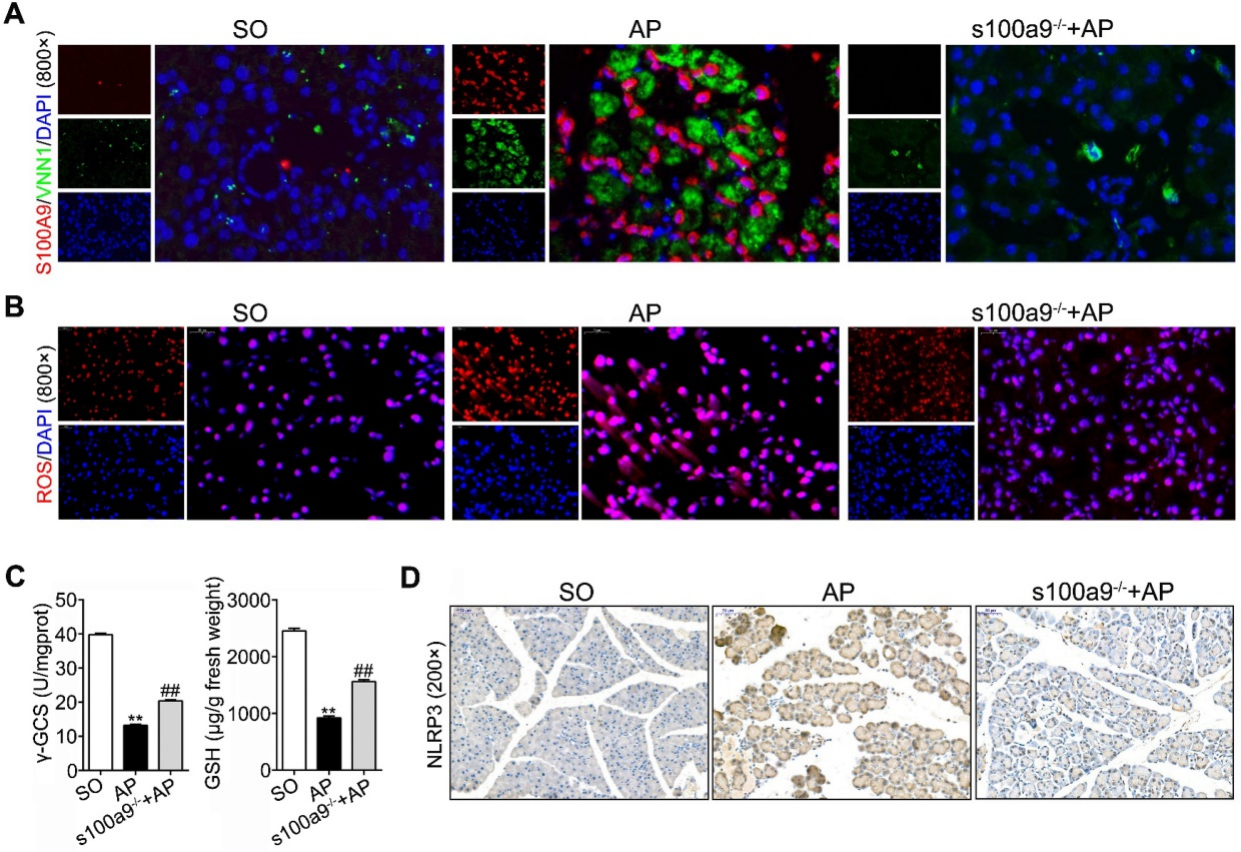
** Loss of S100A9 inhibits NLRP3 activation by decreasing VNN1-mediated ROS release.** (A) Double staining experiments showed that both S100A9 and VNN1 were all upregulated in the pancreatic tissue of AP mice. (B) s100a9^-/-^ mice showed decreased ROS (red fluorescence) release. (C) s100a9^-/-^ mice showed increased γ-GCS and GSH levels (n = 8). (D) s100a9^-/-^ mice showed downregulated NLRP3 expression. Data are presented as the mean ± SEM; **P < 0.01 *vs.* SO mice; ^##^P < 0.01 *vs.* AP mice.

As shown in Figure 6A and Supplementary Figure S5A, IF staining found that both S100A9 (red fluorescence) and VNN1 (green fluorescence) were all increased in AP mice but decreased in s100a9^-/-^ mice. Meanwhile, ROS (red fluorescence) (Figure 6B), and γ-GCS and GSH (Figure 6C) detections were performed, and the results showed that loss of s100a9 can obviously improve oxidative stress in the AP pancreas. The IHC results further proved that NLRP3 protein expression was elevated in AP mice but obviously downregulated in s100a9^-/-^ mice (Figure 6D and Supplementary Figure S5B). These results showed that s100a9 deletion decreases pancreatic injury in AP mice by decreasing NLRP3 activation via inhibition of the VNN1/ROS signaling pathway.

### The S100A9 protein has an interaction with the VNN1 protein

**Figure 7:**
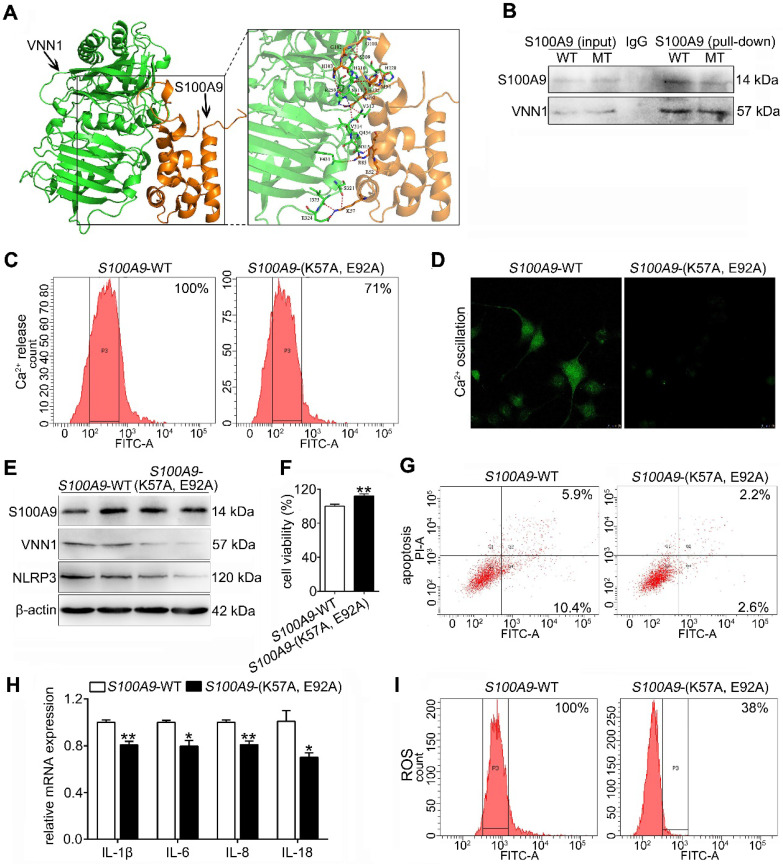
** The S100A9 protein has an interaction with the VNN1 protein.** (A) Binding model between S100A9 protein and VNN1 protein; the surfaces of S100A9 and VNN1 are colored orange and green, respectively. (B) Co-IP results indicated that S100A9 antibody pulled down less VNN1 protein in *S100A9*-MT group than in *S100A9*-WT group. (C-D) Ca^2+^ release and oscillation were all remarkedly downregulated in the MT variant of *S100A9* compared with *S100A9-*WT. (E) *S100A9*-MT plasmid had no obvious effect on S100A9 protein expression but could downregulate VNN1 and NLRP3 protein levels compared with *S100A9-*WT. (F-I) *S100A9*-MT significantly increased cell viability (n = 6), and decreased cell apoptosis, inflammatory factor releases (n = 3) and ROS level of H6C7 cells compared with *S100A9-*WT. Data are presented as the mean ± SEM; *P < 0.05 and **P < 0.01 *vs. S100A9*-WT group.

To investigate the binding mode of the S100A9 and VNN1 proteins, a docking simulation study was carried out, and the weighted score was -740.5. The binding model between S100A9 and VNN1 is shown in Figure 7A and Supplementary Figure S6. The surfaces of S100A9 and VNN1 are colored orange and green, respectively. In summary, as shown in Supplementary Table S4, the docking simulation study indicated that the Glu52, Lys57, Arg85, Glu92, Met94, Gly100, Gly102, His103 and His105 residues in S100A9 are involved in binding with the His228, Arg259, Ser309, His310, Ser311, Val313, Val314, Asn315, Ser321, Ile323, Glu324, Phe431 and Gln434 residues in VNN1 through salt bridges or hydrogen bond interactions. Among these residues, the nitrogen atom of the amino group of Lys57 in S100A9 forms salt bridges with the oxygen atoms of the carboxyl group of Glu324 in VNN1, and the oxygen atom of the carboxyl group of Glu92 in S100A9 forms salt bridges with the nitrogen atoms of the guanidine group of Arg259 in VNN1. Lys57 and Glu92 in S100A9 may play key roles in the interaction between the S100A9 and VNN1 proteins; therefore, we constructed the mutant plasmid of *S100A9*-(K57A, E92A) by mutating both Lys57 (K57) and Glu92 (E92) into Ala (A). Ala (A) is a kind of uncharged amino acid with no heteroatom or benzene ring, and it may result in the loose bonding between S100A9 and VNN1. *S100A9*-(K57A, E92A) is a dominant negative mutant (MT) of *S100A9*-wild type (WT). As shown in Figure 7B, the Co-IP results indicated that the S100A9 pulled down less VNN1 protein in the *S100A9*-(K57A, E92A) group than in the *S100A9*-WT group. Moreover, Ca^2+^ release (Figure 7C) and oscillation (Figure 7D) were all remarkedly downregulated in the MT variant of S100A9 compared with WT-*S100A9*, which proved that the binding between S100A9 and VNN1 requires Ca^2+^ binding. Furthermore, western blotting results showed that *S100A9-*(K57A, E92A) plasmid had no obvious effect on S100A9 protein expression but could downregulate VNN1 and NLRP3 protein levels compared with the *S100A9*-WT plasmid (Figure 7E). In addition, compared to the WT treatment, *S100A9-*(K57A, E92A) improved cell injury by increasing cell viability (Figure 7F) and decreasing cell apoptosis (Figure 7G), inflammatory factor releases (Figure 7H) and ROS level (Figure 7I). These results indicated that S100A9 has an interaction with VNN1 via specific amino acid residues.

### Small molecular inhibitors of S100A9-VNN1 interaction maybe potential anti-AP drugs

**Figure 8:**
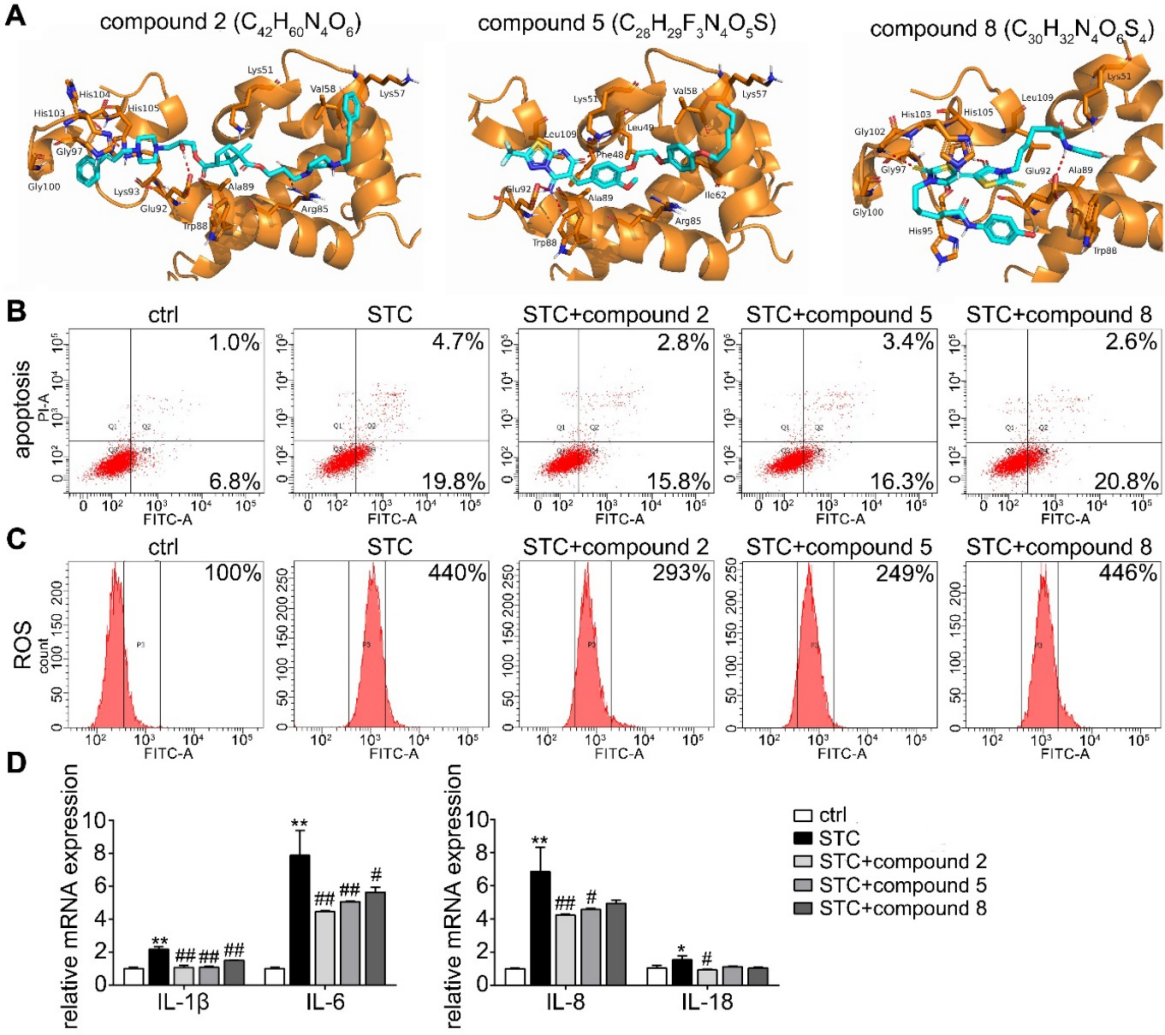
** Effects of small molecular inhibitors of S100A9-VNN1 interaction on STC-induced H6C7 cells injury.** (A) The binding models of compounds 2, 5 and 8 with S100A9 protein. (B-D) Effects of compounds 2, 5 and 8 against STC-induced apoptosis, ROS release and inflammatory response (n = 3). Data are presented as the mean ± SEM; *P < 0.05 and **P < 0.01 *vs.* ctrl group; ^#^P < 0.05 and ^##^P < 0.01 *vs.* STC group.

Based on the interaction between S100A9 and VNN1, the top 100 hits for inhibitors of this interaction were finally selected via the virtual screening (VS) method, and their structures and docking scores are shown in Supplementary Figure S7. Next, the toxicities (Supplementary Figure S8A) and pharmacodynamics (Supplementary Figure S8B) *in vitro* of the top 8 compounds were analyzed, and the results showed that compared to the model treatment, compounds C_42_H_60_N_4_O_6_, C_28_H_29_F_3_N_4_O_5_S and C_30_H_32_N_4_O_6_S_4_ (Chemdiv ID: 0884-0014 (compound 2), 3948-1191 (compound 5) and 3232-0780 (compound 8)) can significantly improve STC-induced cell injury at the maximum nontoxic concentration (Supplementary Figure S8C). The binding modes of these compounds with the S100A9 protein are illustrated in Figure 8A and Supplementary Figure S9, and they all showed suitable steric complementarity with the binding site of S100A9. Briefly, as shown in Supplementary Table S5, C_42_H_60_N_4_O_6_ forms hydrogen bonds with the Arg85, Glu92, His103, Gly97, Gly100, Ala89, Trp88 and Val58 residues in S100A9; C_28_H_29_F_3_N_4_O_5_S forms a salt bridge with the side chain oxygen atom of Glu92 and hydrogen bonds with the Lys51, Trp88, Val58, Ile62, Leu49, Phe48 and Leu109 residues in S100A9; and C_30_H_32_N_4_O_6_S_4_ forms hydrogen bonds with the Glu92, Gly102, His105, Leu109, Als89, Trp88, Gly97 and Gly100 residues in S100A9. These interactions mainly contribute to the binding energy between these compounds and protein S100A9. Moreover, as shown in Figure 8B-D, we found that compounds 2 and 5 have better inhibition effects against STC-induced apoptosis, ROS release and inflammatory response.

**Figure 9:**
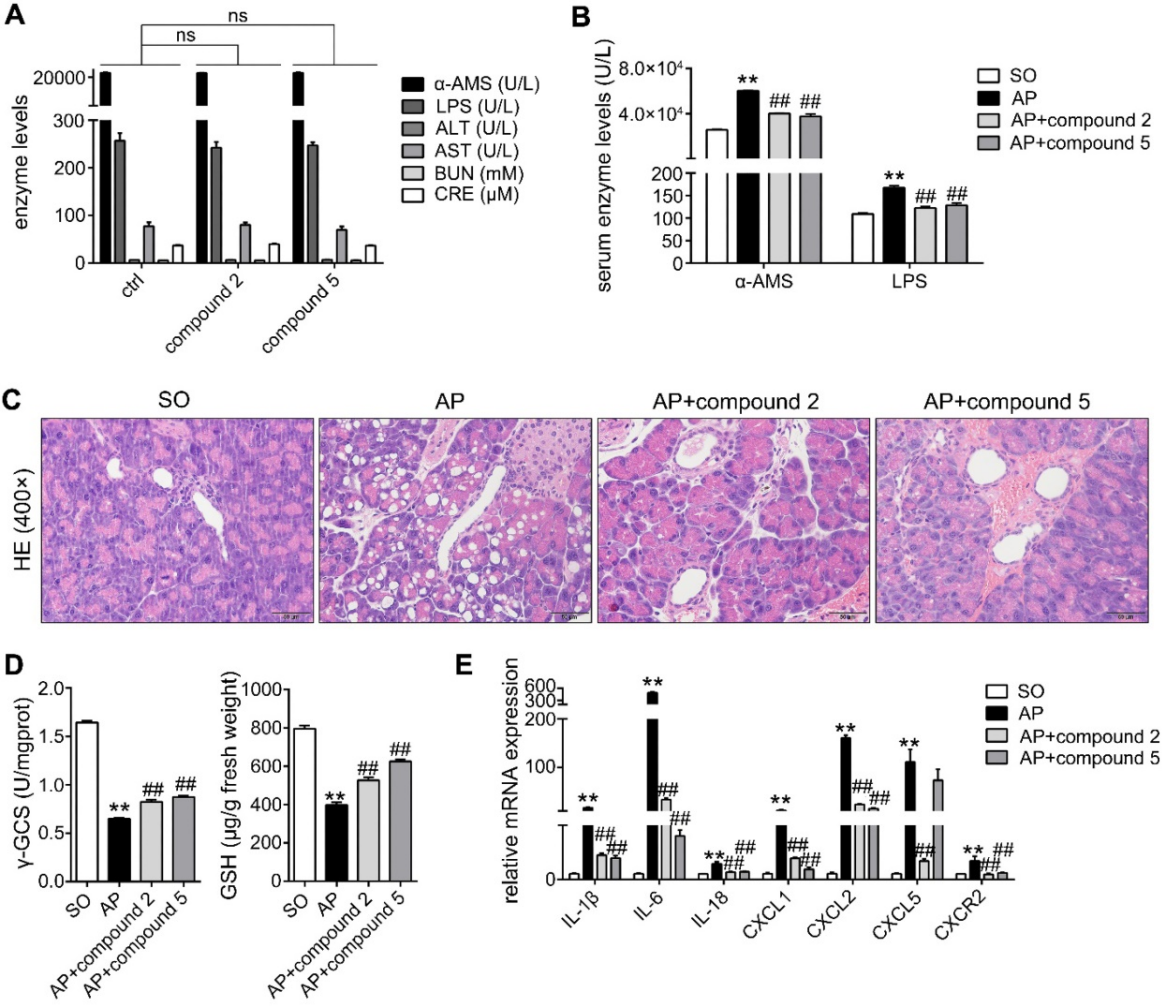
** Effects of small molecular inhibitors of S100A9-VNN1 interaction on AP injury.** (A) Compounds 2 and 5 at the dose of 10 mg/kg/day for 2 days have no obvious pancreatic, hepatic and renal toxicities (n = 6). (B-E) Compounds 2 and 5 at the doses of 10 mg/kg/day for 2 days can significantly inhibit α-AMS and LPS activities (n = 6) and improve AP damage through increasing γ-GCS and GSH (n = 6), and decreasing inflammatory response (n = 3). Data are presented as the mean ± SEM; **P < 0.01 *vs.* SO mice; ^##^P < 0.01 *vs.* AP mice.

In addition, in order to further study the actions of compounds 2 and 5 against AP, we performed the toxicology and pharmacodynamics experiments *in vivo*. As shown in Figure 9A, the results showed that α-AMS and LPS (pancreatic toxicity markers), ALT and AST (hepatotoxicity markers), BUN and CRE (nephrotoxicity markers) levels in serum had no obvious changes in compound groups compared with ctrl group. Moreover, HE staining results of pancreas, heart, liver, spleen, lung, kidney, brain and intestines also proved that compounds 2 and 5 have no marked toxicities at the doses of 10 mg/kg/day for 2 days (Supplementary Figure S10). In addition, compounds 2 and 5 at the doses of 10 mg/kg/day for 2 days can significantly inhibit α-AMS and LPS activities (Figure 9B) and improve AP damage (Figure 9C) through increasing γ-GCS and GSH (Figure 9D), and decreasing inflammatory response (Figure 9E). Therefore, compounds 2 and 5 maybe the potent AP therapeutic drugs.

## Discussion

The overall pathophysiology of pancreatitis involves not only pancreatic acinar cells but also duct cells; however, the contribution of the pancreatic duct to acinar cell homeostasis is unknown, and the molecular mechanisms leading to acinar insult and AP are poorly understood. In this study, we investigated the effects of S100A9 in the pancreatic duct and how S100A9 affects AP development. First, we constructed a coculture system for pancreatic duct cells and acinar cells and found that injured H6C7 cells can induce an increased apoptosis rate and reduced exocrine function in primary acinar cells. In this process, we found that release of inflammatory factors, including IL-1β, IL-6, IL-8 and IL-18, and NLRP3 expression, were significantly increased in H6C7 cells. NLRP3 is the most wellcharacterized inflammasome sensor molecule and is associated with a diverse range of diseases and conditions, including but not limited to Alzheimer's disease, atherosclerosis, gout and type 1 diabetes 31. NLRP3 inflammasome can result in the recruitment and activation of pro-caspase-1, which cleaves pro-IL-1β into IL-1β and actives gasdermin D to unleash its pore-forming N-terminal domain 32,33. Recent studies have shown that the NLRP3 inflammasome regulates the development of SIRS and compensatory anti-inflammatory response syndromes (CARS) in AP mice, and inhibition of NLRP3 activation may be a useful treatment for AP patients 34-36.

S100A8 and S100A9 are Ca^2+^-binding proteins belonging to the S100 family, and they always exist in the form of heterodimer 37. ELISA results showed that single S100A8 and S100A9 levels were all significantly increased, but the S100A8/9 heterodimer level has no obvious change in STC-injured H6C7 cells. To further verify the effect of S100A9 on pancreatic duct cell damage in AP, we respectively knocked down and overexpressed the S100A9 protein in H6C7 cells. Knockdown of S100A9 can significantly decrease late apoptosis and inflammatory factors, and overexpression of S100A9 can notably increase cell injury by regulating NLRP3 expression. S100A9 is known to be a ligand for TLR4 and RAGE, to stimulate pro-inflammatory responses 38. We used Paq and FPS-ZM1 to inhibit S100A9-mediated TLR4 and RAGE signaling and found that S100A9 overexpression also significantly up-regulated inflammation compared to NC group, which proved that there may exist other inflammatory signaling pathway independent of these two known receptors. Therefore, we carried out an IP experiment to identify the interacting proteins of S100A9, and the results showed that S100A9 can pull down the VNN1 protein, which was also verified by the Co-IP experiment. Moreover, knockdown and overexpression of S100A9 accordingly changed VNN1 expression, which proved that VNN1 may be a downstream target of S100A9. VNN1 is a GPI-anchored ectoenzyme which hydrolyzes pantetheine to pantothenic acid and cysteamine, which can inhibit GSH and γ-GCS activities. Previous studies have focused on the ability of VNN1 to act as a key tissue sensor for oxidative stress and proved that upregulation of VNN1 levels results in an increased inflammation in the microenvironment and poor resistance to oxidative stress 39-41. In STC-injured H6C7 cells, cysteamine, GSH, γ-GCS and ROS levels were all significantly increased through up-regulation of VNN1 expression instead of its enzymatic activity. Subsequently, we knocked down VNN1 expression after overexpressing S100A9 in H6C7 cells, and the results showed that inhibition of VNN1 can reverse S100A9 overexpression-induced cell injury and inflammation; this effect may be involved in decreasing ROS release via upregulation of γ-GCS and GSH levels. Furthermore, S100A9 is a secretory protein, and it can be secreted out of the cells from cytoplasm 42. In order to judge which part of S100A9 works with VNN1, we constructed a dep-*S100A9* plasmid, whose S100A9 protein secretion outside of the cell was significantly decreased. Our results showed that VNN1 expression, inflammatory factors, cell apoptosis and ROS are also notably decreased in dep-*S100A9* group compared with *S100A9* group. Moreover, considering that VNN1 is GPI-anchored ectoenzyme, these results proved that S100A9 exerts its actions mainly through the extracellular S100A9.

*In vivo,* s100a9^-/-^ C57BL/6 mice were also constructed, and the pancreas showed milder injury and lower serum enzyme (α-AMS and LPS) and inflammatory levels compared to those in the AP pancreas. Moreover, ROS was notably downregulated in s100a9^-/-^ pancreas, and the molecular mechanism involved in the inhibition of NLRP3 activation by decreasing VNN1-mediated ROS production. Therefore, S100A9 upregulation may increase NLRP3 activation by inducing VNN-1-mediated ROS release during pancreatic duct injury in AP, which verifies that S100A9 is a critical target for AP therapy.

In addition, we discussed the interaction between S100A9 and VNN1 and found that Lys57 and Glu92 of the S100A9 protein can form salt bridges with Glu324 and Arg259 of the VNN1 protein, respectively. Therefore, we constructed the mutant plasmid *S100A9*-(K57A, E92A) by mutating both Lys57 and Glu92 into Ala. The study showed that the MT-S100A9 plasmid significantly downregulated VNN1 expression, inflammatory factor releases and ROS levels compared to those observed for WT-*S100A9,* which indicated that S100A9 has an interaction with VNN1 via specific amino acid residues. To achieve more precise targeting and less toxicity, we further screened potential inhibitors of S100A9-VNN1, ranked the top 100 hits from 100,000 compounds in Chemdiv based on these binding sites and evaluated the *in vitro* pharmacodynamics of 8 hit compounds. The results indicated that compounds C_42_H_60_N_4_O_6_ and C_28_H_29_F_3_N_4_O_5_S can significantly improve AP injury *in vitro* and *in vivo*; these compounds may be potent inhibitors of the S100A9-VNN1 interaction and serve as AP therapeutic drugs with less toxicity.

## Conclusions

In summary, the present paper showed the important regulatory effect of S100A9 in pancreatic duct injury in AP, and inhibition of the S100A9-VNN1 interaction may be a key therapeutic target for this disease. Furthermore, a variety of small molecular compounds based on this key therapeutic target for AP were screened and verified, which provides an experimental basis for the clinical treatment of AP in the future.

## Supplementary Material

Supplementary figures and tables.

## Abbreviations

γ-GCS: γ-glutamylcysteine synthetase
ADM: acinar-to-ductal metaplasia
AMS: amylase
AP: acute pancreatitis
CARS: compensatory anti-inflammatory response syndromes
ELISA: enzyme linked immunosorbent assay
GSH: glutathione
HPLC: high-performance liquid chromatography
IF: immunofluorescence
IHC: immunohistochemistry
LPS: lipase
MRP14: myeloid-related protein 14
NLRP3: NLR family, pyrin domain containing 3
PDAC: pancreatic ductal adenocarcinoma
RAGE: receptor of advanced glycation endproducts
S100A9: S100 calcium binding protein A9
SAP: severe acute pancreatitis
SIRS: systemic inflammatory response syndrome
STC: sodium taurocholate
TLR4: toll-like receptor 4
VNN1: vanin1

## References

[1] van Dijk SM, Hallensleben NDL, van Santvoort HC, Fockens P, van Goor H, Bruno MJ (2017). Acute pancreatitis: recent advances through randomised trials. Gut.

[2] Tenner S, Baillie J, DeWitt J, Vege SS, American College of Gastroenterology (2013). American College of Gastroenterology guideline: management of acute pancreatitis. Am J Gastroenterol.

[3] Lankisch PG, Ape M, Banks PA, Acute pancreatitis Lancet. 2015; 386: 85-96.

[4] Trikudanathan G, Wolbrink DRJ, van Santvoort HC, Mallery S, Freeman M, Besselink MG (2019). Current concepts in severe acute and necrotizing pancreatitis: an evidence-based approach. Gastroenterology.

[5] Hines QJ, Pandol SJ (2019). Management of severe acute pancreatitis. BMJ.

[6] Wilmer A (2004). ICU management of severe acute pancreatitis. Eur J Intern Med.

[7] Tan JH, Cao RC, Zhou L, Zhou ZT, Chen HJ, Xu J (2020). ATF6 aggravates acinar cell apoptosis and injury by regulating p53/AIFM2 transcription in severe acute pancreatitis. Theranostics.

[8] Quilichini E, Fabre M, Dirami T, Stedman A, De Vas M, Ozguc O (2019). Pancreatic ductal deletion of Hnf1b disrupts exocrine homeostasis, leads to pancreatitis, and facilitates tumorigenesis. Cell Mol Gastroenterol Hepatol.

[9] Zeng M, Szymczak M, Ahuja M, Zheng CY, Yin HE, Swaim W (2017). Restoration of CFTR activity in ducts rescues acinar cell function and reduces inflammation in pancreatic and salivary glands of mice. Gastroenterology.

[10] Murtaugh LC, Keefe MD (2015). Regeneration and repair of the exocrine pancreas. Annu Rev Physiol.

[11] Pandol SJ, Saluja AK, Imrie CW, Banks PA (2007). Acute pancreatitis: bench to the bedside. Gastroenterology.

[12] Pallagi P, Balla Z, Singh AK, Dósa S, Iványi B, Kukor Z (2014). The role of pancreatic ductal secretion in protection against acute pancreatitis in mice. Crit Care Med.

[13] Reichert M, Rustgi AK (2011). Pancreatic ductal cells in development, regeneration, and neoplasia. J Clin Invest.

[14] Molnar R, Madácsy T, Varga A, Németh M, Katona X, Görög M (2020). Mouse pancreatic ductal organoid culture as a relevant model to study exocrine pancreatic ion secretion. Lab Invest.

[15] Pallagi P, Hegyi P, Rakonczay ZJ (2015). The physiology and pathophysiology of pancreatic ductal secretion: the background for clinicians. Pancreas.

[16] Xiang H, Zhang QK, Wang DQ, Xia SL, Wang GJ, Zhang GX (2016). iTRAQ-based quantitative proteomic analysis for identification of biomarkers associated with emodin against severe acute pancreatitis in rats. RSC Adv.

[17] Wang S, Song R, Wang Z, Jing Z, Wang S, Ma J (2018). S100A8/A9 in inflammation. Front Immunol.

[18] Marinkovic G, Koenis DS, de Camp L, Jablonowski R, Graber N, de Waard V (2020). S100A9 links inflammation and repair in myocardial infarction. Circ Res.

[19] Ulas T, Pirr S, Fehlhaber B, Bickes MS, Loof TG, Vogl T (2017). S100-alarmin-induced innate immune programming protects newborn infants from sepsis. Nat Immunol.

[20] Chen KT, Kim PD, Jones KA, Devarajan K, Patel BB, Hoffman JP (2014). Potential prognostic biomarkers of pancreatic cancer. Pancreas.

[21] Schnekenburger J, Schick V, Krüger B, Manitz MP, Sorg C, Nacken W (2008). The calcium binding protein S100A9 is essential for pancreatic leukocyte infiltration and induces disruption of cell-cell contacts. J Cell Physiol.

[22] Farkas GJ, Tiszlavicz Z, Takács T, Szabolcs A, Farkas G, Somogyvári F (2014). Analysis of plasma levels and polymorphisms of S100A8/9 and S100A12 in patients with acute pancreatitis. Pancreas.

[23] Tao XF, Chen Q, Li N, Xiang H, Pan Y, Qu YY (2020). Serotonin-RhoA/ROCK axis promotes acinar-to-ductal metaplasia in caerulein-induced chronic pancreatitis. Biomed Pharmacother.

[24] Kuśmierek K, Bald E (2008). Measurement of reduced and total mercaptamine in urine using liquid chromatography with ultraviolet detection. Biomed Chromatogr.

[25] Ji Y, Luo H, Li H, Lin Z, Luo W (2020). Determination of plasma homocysteine with a UHPLC-MS/MS method: application to analyze the correlation between plasma homocysteine and whole blood 5-methyltetrahydrofolate in healthy volunteers. Biomed Chromatogr.

[26] Madhi R, Rahman M, Mörgelin M (2019). c-Abl kinase regulates neutrophil extracellular trap formation, inflammation, and tissue damage in severe acute pancreatitis. J Leukoc Biol.

[27] Kozakov D, Beglov D, Bohnuud T, Mottarella SE, Xia B, Hall DR (2013). How good is automated protein docking?. Proteins.

[28] Kozakov D, Hall DR, Xia B, Porter KA, Padhorny D, Yueh C (2017). The ClusPro web server for protein-protein docking. Nat Protoc.

[29] Molecular Operating Environment (MOE), 2018.01, Chemical Computing Group Inc, 1010 Sherbooke St West, Suite #910, Montreal, QC, Canada, H3A 2R7. 2018.

[30] Molecular Operating Environment (MOE), 2015.10, Chemical Computing Group Inc, 1010 Sherbooke St West, Suite #910, Montreal, QC, Canada, H3A 2R7. 2015.

[31] Mangan MSJ, Olhava EJ, Roush WR, Seidel HM, Glick GD, Latz E (2018). Targeting the NLRP3 inflammasome in inflammatory diseases. Nat Rev Drug Discov.

[32] Li Z, Guo J, Bi L (2020). Role of the NLRP3 inflammasome in autoimmune diseases. Biomed Pharmacother.

[33] Tschopp J, Schroder K (2010). NLRP3 inflammasome activation: the convergence of multiple signalling pathways on ROS production?. Nat Rev Immunol.

[34] Sendler M, van den Brandt C, Glaubitz J, Wilden A, Golchert J, Weiss FU (2020). NLRP3 inflammasome regulates development of systemic inflammatory response and compensatory anti-inflammatory response syndromes in mice with acute pancreatitis. Gastroenterology.

[35] Fu Q, Zhai ZS, Wang YZ, Xu LX, Jia PC, Xia P (2019). NLRP3 deficiency alleviates severe acute pancreatitis and pancreatitis-associated lung injury in a mouse model. Biomed Res Int.

[36] Jin HZ, Yang XJ, Zhao KL, Mei FC, Zhou Y, You YD (2019). Apocynin alleviates lung injury by suppressing NLRP3 inflammasome activation and NF-kappaB signaling in acute pancreatitis. Int Immunopharmacol.

[37] Pruenster M, Vogl T, Roth J, Sperandio M (2016). S100A8/A9: from basic science to clinical application. Pharmacol Ther.

[38] Kovačić M, Mitrović-Ajtić O, Beleslin-Čokić B, Djikić D, Subotički T, Diklić M (2018). TLR4 and RAGE conversely mediate pro-inflammatory S100A8/9-mediated inhibition of proliferation-linked signaling in myeloproliferative neoplasms. Cell Oncol (Dordr).

[39] Kang MX, Qin WJ, Buya M, Dong X, Zheng W, Lu WJ (2016). VNN1, a potential biomarker for pancreatic cancer-associated new-onset diabetes, aggravates paraneoplastic islet dysfunction by increasing oxidative stress. Cancer Lett.

[40] Berruyer C, Martin FM, Castellano R, Macone A, Malergue F, Garrido-Urbani S (2004). Vanin-1-/- mice exhibit a glutathione-mediated tissue resistance to oxidative stress. Mol Cell Biol.

[41] Berruyer C, Pouyet L, Millet V, Martin FM, LeGoffic A, Canonici A (2006). Vanin-1 licenses inflammatory mediator production by gut epithelial cells and controls colitis by antagonizing peroxisome proliferator-activated receptor gamma activity. J Exp Med.

[42] Lee Y, Jang S, Min JK, Lee K, Sohn KC, Lim JS (2012). S100A8 and S100A9 are messengers in the crosstalk between epidermis and dermis modulating a psoriatic milieu in human skin. Biochem Biophys Res Commun.

