# Supplementary material for: Pancreatic ductal deletion of S100A9 alleviates acute pancreatitis by targeting VNN1-mediated ROS release to inhibit NLRP3 activation
Source: Theranostics. 2021 Mar 4;11(9):4467–82. doi: 10.7150/thno.54245 (PMC7977474; doi:10.7150/thno.54245)
Supplement: Supplementary file 1 — Supplementary figures and tables. [file thnov11p4467s1.pdf]

Supplementary material for

Pancreatic ductal deletion of S100A9 alleviates acute pancreatitis by targeting VNN1-mediated ROS release to inhibit NLRP3 activation

Hong Xiang[1,*], Fangyue Guo[1,2,*], Xufeng Tao[3,*], Qi Zhou[1,2], Shilin Xia[1], Dawei Deng[4], Lunxu Li[4], Dong Shang[1,2,4,#]

[1]Laboratory of Integrative Medicine, First Affiliated Hospital of Dalian Medical University, Dalian, 116011, China;
[2]Institute (College) of Integrative Medicine, Dalian Medical University, Dalian, 116044, China;
[3]School of Chemical Engineering, Dalian University of Technology, Dalian, 116024, China;
[4]Department of General Surgery, First Affiliated Hospital of Dalian Medical University, Dalian, 116011, China.

* These authors contributed equally to this work.
[#] Corresponding Author: Dong Shang, Laboratory of Integrative Medicine, First Affiliated Hospital of Dalian Medical University, Dalian, 116011, China; shangdong@dmu.edu.cn

Supplementary Table S1 Primer sequences used for qPCR assay

Gene	species	Primers (5'-3')
β-actin	Human	Forward: CCTGGGCATGGAGTCCTGTG<br>Reverse: TCTTCATTGTGCTGGGTGCC
IL-1β	Human	Forward: ATGATGGCTTATTACAGTGGCAA<br>Reverse: GTCGGAGATTCGTAGCTGGA
IL-6	Human	Forward: AAGCCAGAGCTGTGCAGATGAGTA<br>Reverse: TGTCCTGCAGCCACTGGTTC
IL-8	Human	Forward: CACTGTGTGTAAACATGACTTCCAA<br>Reverse: TGTGGTCCACTCTCAATCACTCTC
IL-18	Human	Forward: TCTTCATTGACCAAGGAAATCGG<br>Reverse: TCCGGGGTGCATTATCTCTAC
S100A8	Human	Forward: AAATTGCTAGAGACCGAGTGTCCT<br>Reverse: CACGCCCATCTTTATCACCA
S100A9	Human	Forward: TCCACCAATACTCTGTGAAGCTG<br>Reverse: CCTCCATGATGTGTTCTATGACC
HSPA1B	Human	Forward: GGTCAGGCCCTACCATTGAG<br>Reverse: TCCTTGAGTCCCAACAGTCCA
HSPA8	Human	Forward: AAGCTATGTCGCCTTTACGGACAC<br>Reverse: CATCATCAAATCTGCGTCCAATC
HNRNPH2	Human	Forward: ACCGTCTCGCTATAGCCGTTTG<br>Reverse: CATCACTTCATCGGCTGAGCA
PABPC1	Human	Forward: GGTTCCAAGGGCTATGGATTTGTA<br>Reverse: GCCCTAGCTCCAAGTTCAGCTTC
TUBB4A	Human	Forward: CCATGGACTCTGTCCGTTCT<br>Reverse: CCCTTTGCCCAGTTGTTG
TUBA1C	Human	Forward: CTGAGCAACTCATCACAGGCAAG<br>Reverse: CCATGAGCAGCGAGGTGAAC
TBC1D2	Human	Forward: CAAGACTCCCAGCCGGGTTA<br>Reverse: GGAATTCCCAGCGCTTCATC
VNN1	Human	Forward: TGGCACTTTCGGAACCCAGTA<br>Reverse: TCAGACTAAACAAGCGTCCGTCAG
β-actin	Mouse	Forward: CATCCGTAAAGACCTCTATGCCAAC<br>Reverse: ATGGAGCCACCGATCCACA
IL-1β	Mouse	Forward: AAATCTCGCAGCAGCACATCAA<br>Reverse: CCACGGGAAAGACACAGGTAGC
IL-6	Mouse	Forward: CCTTCTTGGGACTGATGATGCTG<br>Reverse: TTGGGAGTGGTATCCTCTGTGA
IL-18	Mouse	Forward: CAGGCCTGACATCTTCTGCAA<br>Reverse: TCTGACATGGCAGCCATTGT
CXCL1	Mouse	Forward: ATGGCTGGGATTCACCTCAAGAAC<br>Reverse: AGTGTGGCTATGACTTCGGTTTGG
CXCL2	Mouse	Forward: CACTGGTCCTGCTGCTGCTG<br>Reverse: GCGTCACACTCAAGCTCTGGATG
CXCL5	Mouse	Forward: ATCCCCAGCGGTTCCATCTCG<br>Reverse: CGTTGCGGCTATGACTGAGGAAG
CXCR2	Mouse	Forward: CAGCTGCCTTAACCCCATC<br>Reverse: CTTGAGAAGTCCATGGCGAAA

Supplementary Table S2 Statistical data of protein identification

Samples	Total spectrogram number	Number of identification spectrogram	Identification of peptide number	Identification of protein number	Unique-2
IgG	4713	493	154	56	32
IP	15232	420	80	20	13

Supplementary Table S3 Unique proteins of pull-down in S100A9 IP group

Accession	Names	Length	Mass	Unused	Coverage(%)
sp|P0DMV9|HS71B_HUMAN	Heat shock 70 kDa protein 1B	641	70051.60	3.33	9.20
sp|P11142|HSP7C_HUMAN	Heat shock cognate 71 kDa protein	646	70897.60	14.69	13.78
sp|P55795|HNRH2_HUMAN	Heterogeneous nuclear ribonucleoprotein H2	449	49229.30	2.00	3.79
sp|P11940|PABP1_HUMAN	Polyadenylate-binding protein 1	636	70670.40	10.43	9.43
sp|P04350|TBB4A_HUMAN	Tubulin beta-4A chain	444	49585.50	2.00	2.70
sp|Q9BQE3|TBA1C_HUMAN	Tubulin alpha-1C chain	449	49894.90	2.08	4.45
sp|Q9BYX2|TBD2A_HUMAN	TBC1 domain family member 2A	928	105412.80	6.14	4.31
sp|O95497|VNN1_HUMAN	Pantetheinase	513	57011.20	2.28	4.09
sp|P04432|KVD39_HUMAN	Immunoglobulin kappa variable 1D-39	117	12737.30	2.00	13.68
sp|P0DOX7|IGK_HUMAN	Immunoglobulin kappa light chain	214	23378.90	2.00	8.41
sp|P01860|IGHG3_HUMAN	Immunoglobulin heavy constant gamma 3	377	41286.60	7.24	15.38

Supplementary Table S4 Contact list between S100A9 and VNN1

The residues in S100A9	The residues in VNN1	Interaction type
Oxygen atom of carboxyl group of Glu92	Nitrogen atoms of guanidine group of Arg259	Salt bridge
Nitrogen atom of amino group of Lys57	Oxygen atoms of carboxyl group of Glu324	Salt bridge
Sulfur atom of thioether group of Met94	Nitrogen atom of imidazolyl group of His228	Hydrogen bond
Oxygen atom of backbone of Gly100	Oxygen atom of hydroxyl group of Ser309	Hydrogen bond
Nitrogen atom of backbone of Gly102	Oxygen atom of hydroxyl group of Ser309	Hydrogen bond
Oxygen atom of backbone of Glu92	Nitrogen atom of imidazolyl group of His310	Hydrogen bond
Nitrogen atom of imidazolyl group of His105	Oxygen atom of backbone of His310	Hydrogen bond
Nitrogen atom of imidazolyl group of His103	Oxygen atom of hydroxyl group of Ser311	Hydrogen bond
Oxygen atom of carboxyl group of Glu92	Carbon atom of backbone of Val313	Hydrogen bond
Oxygen atom of carboxyl group of Glu92	Nitrogen atom of backbone of Val314	Hydrogen bond
Oxygen atom of carboxyl group of Glu52	Nitrogen atom of amido group of Asn315	Hydrogen bond
Carbon atom of sidechain of Lys57	Oxygen atom of backbone of Ser321	Hydrogen bond
Nitrogen atom of amino group of Lys57	Oxygen atom of backbone of Ile323	Hydrogen bond
Nitrogen atom of guanidine group of Arg85	Oxygen atom of backbone of Phe431	Hydrogen bond
Nitrogen atom of guanidine group of Arg85	Oxygen atom of amido group of Gln434	Hydrogen bond

Supplementary Table S5 Contact list between three compounds and S100A9

Compounds	Chemical groups in compound	The residues in S100A9	Interaction type
$C_{42}H_{60}N_4O_6$	Oxygen atom in one hydroxyl group (hydrogen bond acceptor)	Side chain nitrogen atom of Arg85	Hydrogen bond
	Oxygen atom in one hydroxyl group (hydrogen bond donor)	Side chain oxygen atom of Glu92	Hydrogen bond
	Carbon atom (hydrogen bond donor)	Backbone oxygen atom of His103	Hydrogen bond
	Others	Gly97, Gly100, Ala89, Trp88 and Val58	Hydrogen bond
$C_{28}H_{29}F_3N_4O_5S$	Oxygen atom (hydrogen bond acceptor)	Side chain nitrogen atom of Lys51	Hydrogen bond
	Nitrogen atom (hydrogen bond donor)	Backbone oxygen atom of Trp88	Hydrogen bond
	Nitrogen atom	Side chain oxygen atom of Glu92	Salt bridge
	Others	Val58, Ile62, Leu49, Phe48 and Leu109	Hydrogen bond
$C_{30}H_{32}N_4O_6S_4$	Nitrogen atom (hydrogen bond donor)	Side chain oxygen atom of Glu92	Hydrogen bond
	Sulfur atom (hydrogen bond acceptor)	Backbone nitrogen atom of Gly102	Hydrogen bond
	Oxygen atom (hydrogen bond acceptor)	Backbone nitrogen atom of His105	Hydrogen bond
	Others	Leu109, Ala89, Trp88, Gly97 and Gly100	Hydrogen bond

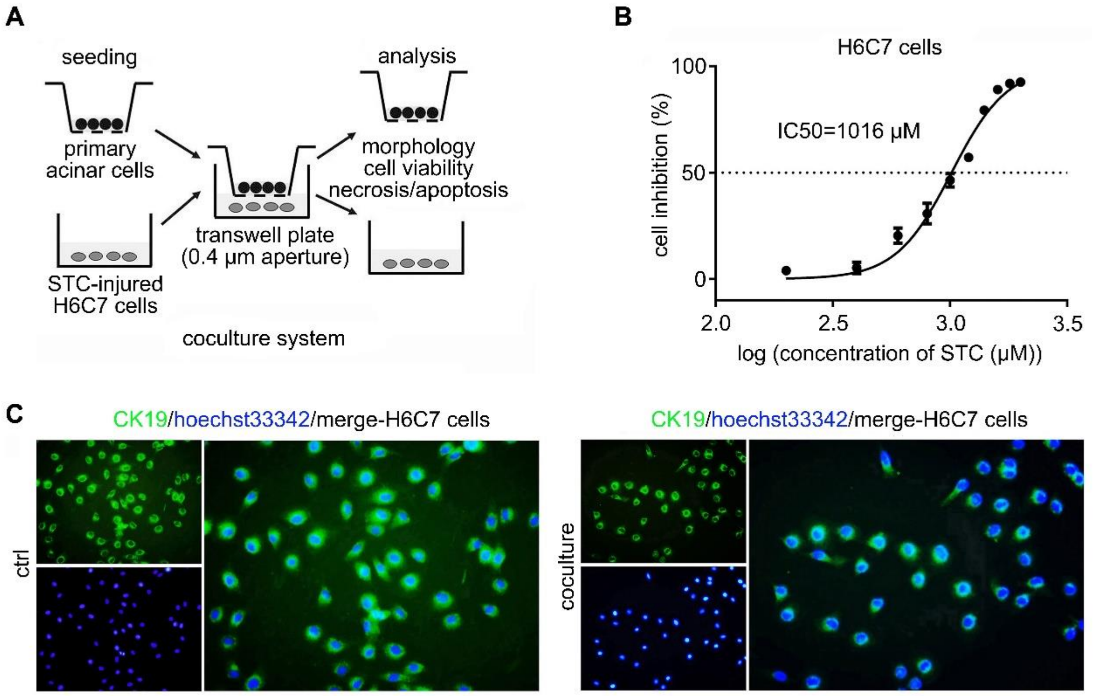

Supplementary Figure S1 (A) Pattern diagram of the coculture system for STC-injured ductal cells and primary acinar cells. (B) MTT results showed that the IC50 value of STC in H6C7 cells was 1016 μM (n = 6). (C) IF staining proved that the expression of the ductal cell marker CK19 clearly decreased in STC-treated H6C7 cells. Data are presented as the mean ± SEM.

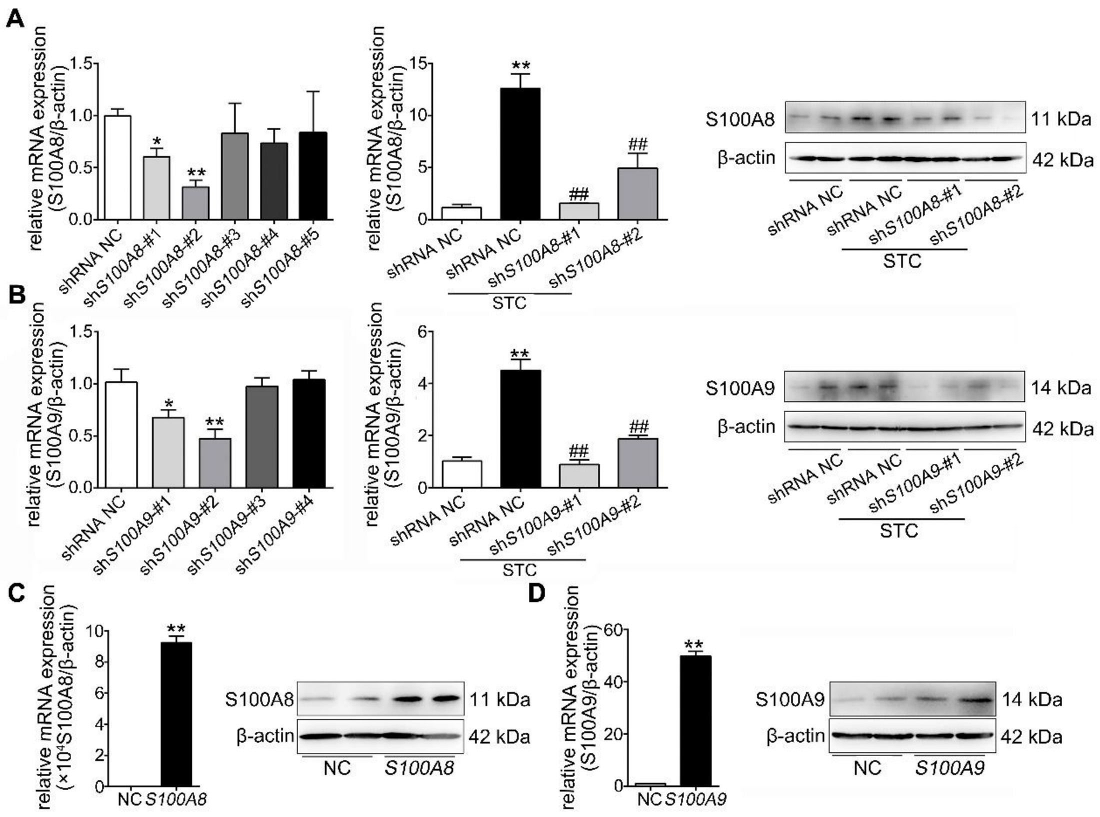

Supplementary Figure S2 (A) sh*S100A8*-#1 and -#2 significantly downregulated S100A8 mRNA (n = 3) and protein expressions. (B) sh*S100A9*-#1 and -#2 significantly downregulated S100A9 mRNA (n = 3) and protein expressions. (C) S100A8 mRNA and protein expression levels were upregulated in S100A8-overexpressing H6C7 cells (n = 3). (D) S100A9 mRNA and protein expression levels were upregulated in S100A9-overexpressing H6C7 cells (n = 3). Data are presented as the mean ± SEM; *P < 0.05 and **P < 0.01 *vs.* shRNA NC or NC group; $^{\#\#}$P < 0.01 *vs.* shRNA NC+STC.

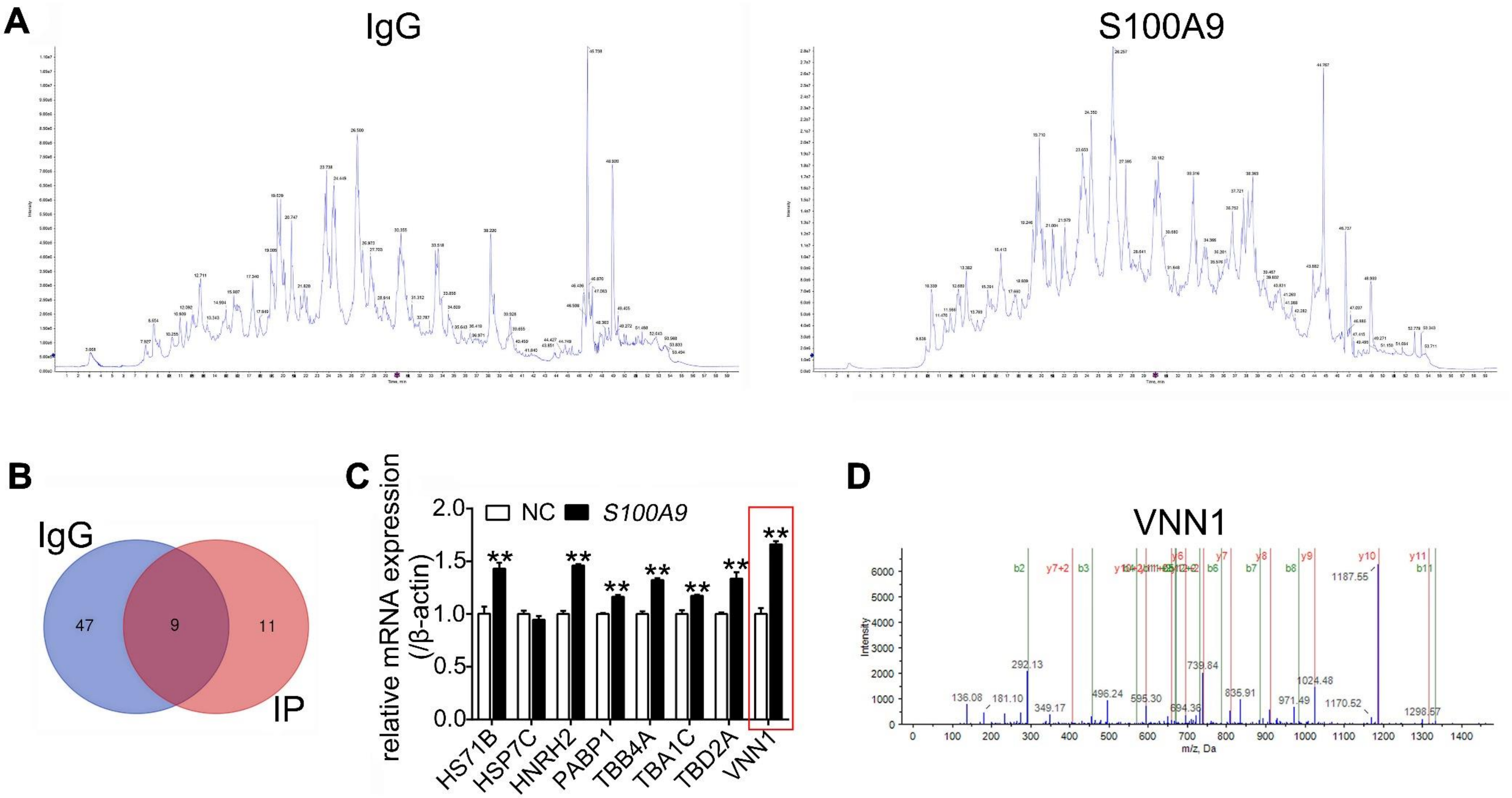

Supplementary Figure S3 (A) LC-MS/MS was performed to identify and analyze the binding proteins of S100A9. (B) 56 and 20 kinds of proteins were identified in the IgG and S100A9 pull-down samples, respectively. (C) Gene expression levels of 8 proteins (HS71B, HSP7C, HNRH2, PABP1, TBB4A, TBA1C, TBD2A and VNN1) in S100A9-overexpressing H6C7 cells were detected by qPCR (n = 3). (D) Mass spectrogram of the VNN1 protein. Data are presented as the mean ± SEM; **P < 0.01 *vs.* NC group.

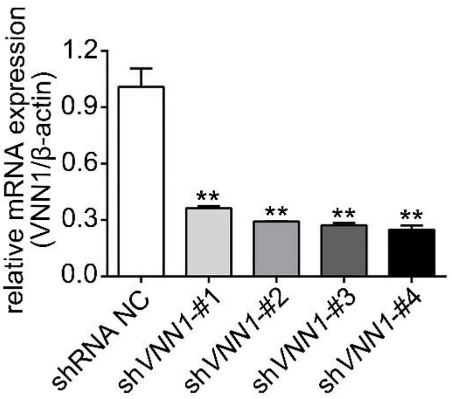

Supplementary Figure S4 sh*VNN1*s significantly downregulated S100A9 mRNA level compared to shRNA NC group (n = 3). Data are presented as the mean ± SEM; $^{**}P < 0.01$ *vs.* shRNA NC group.

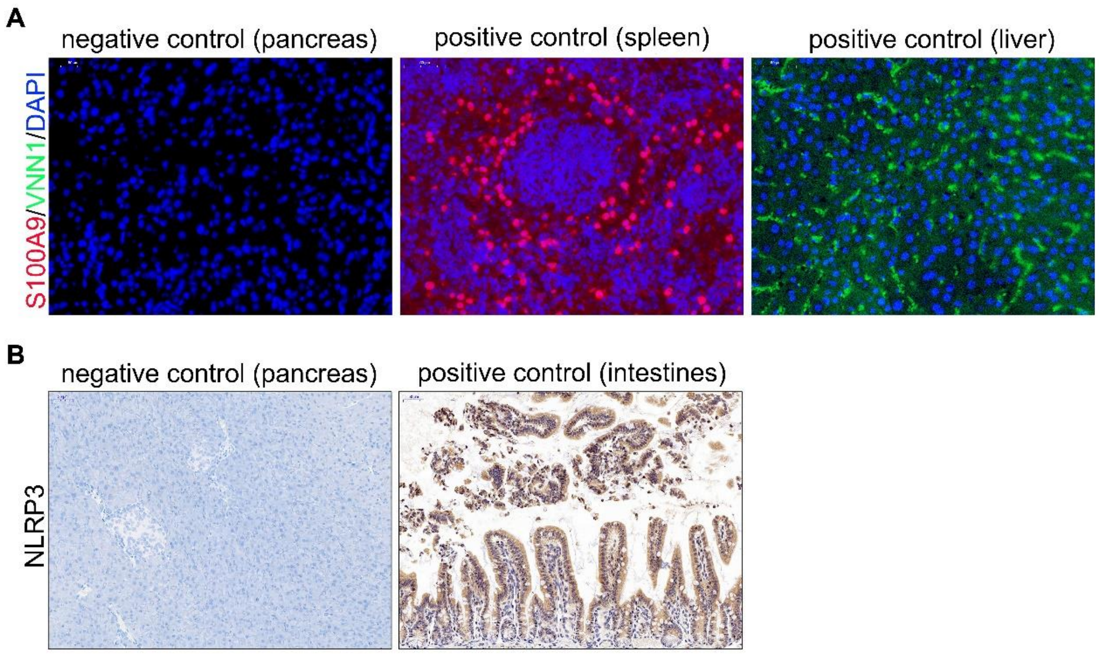

Supplementary Figure S5 (A-B) Negative and positive controls of S100A9, VNN1 and NLRP3 expressions.

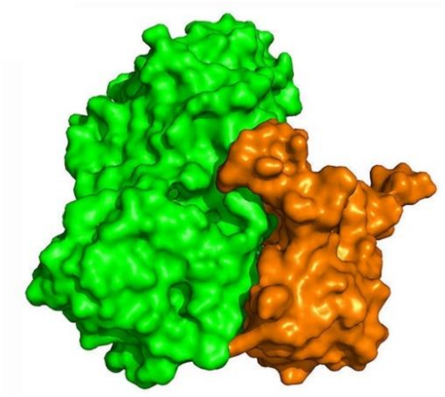

Supplementary Figure S6 Binding model between the S100A9 protein and the VNN1 protein; the surfaces of S100A9 and VNN1 are colored orange and green, respectively.

0683-0021<br>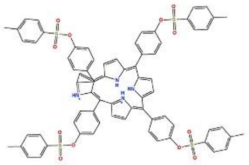<br>S:-10.8913	0884-0014<br>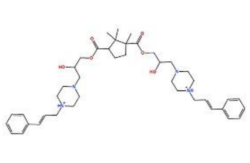<br>S:-8.5406	2372-3991<br>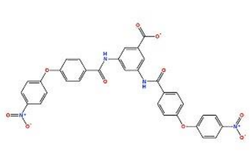<br>S:-8.2299	3948-1149<br>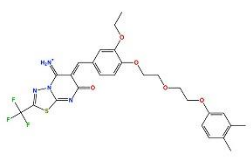<br>S:-8.2043
3948-1191<br>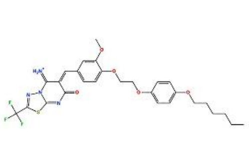<br>S:-8.1575	0249-0003<br>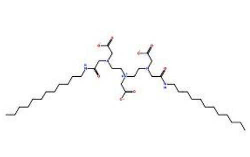<br>S:-8.0931	2324-0140<br>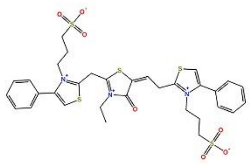<br>S:-8.0680	3232-0780<br>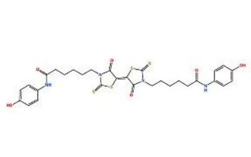<br>S:-8.0591
3289-7235<br>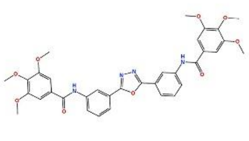<br>S:-8.0092	3861-0047<br>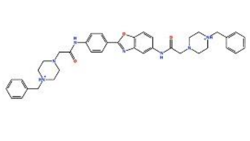<br>S:-7.9737	3797-0694<br>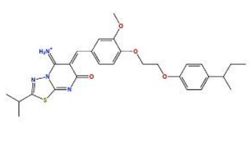<br>S:-7.9408	3537-5285<br>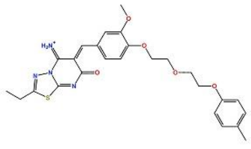<br>S:-7.9036
0927-0035<br>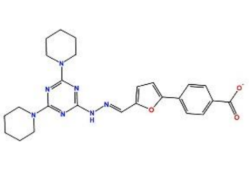<br>S:-7.8888	2040-0304<br>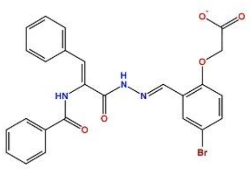<br>S:-7.8013	3232-0776<br>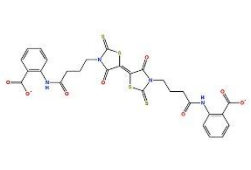<br>S:-7.7541	0669-0140<br>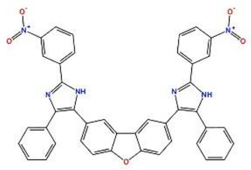<br>S:-7.7270
4011-0808<br>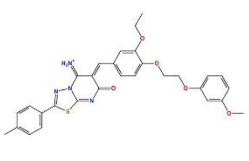<br>S:-7.7152	2036-0830<br>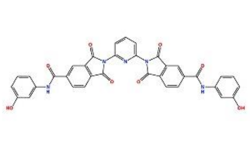<br>S:-7.6875	3948-0977<br>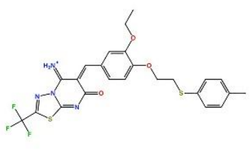<br>S:-7.6810	2324-0157<br>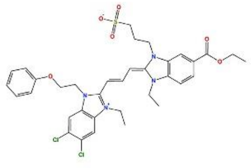<br>S:-7.6803

2185-0036<br>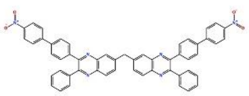<br>S:-7.6740	3894-0003<br>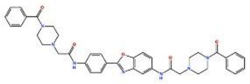<br>S:-7.6408	2324-0151<br>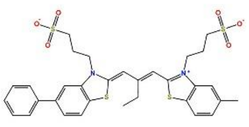<br>S:-7.6389	2030-0071<br>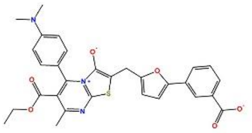<br>S:-7.6349
3948-1099<br>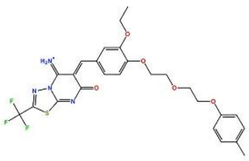<br>S:-7.5964	3948-1042<br>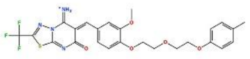<br>S:-7.5867	2033-0102<br>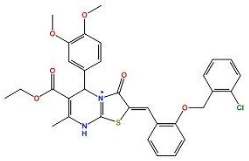<br>S:-7.5851	3819-1354<br>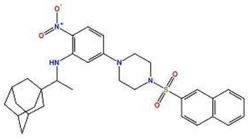<br>S:-7.5820
0105-0143<br>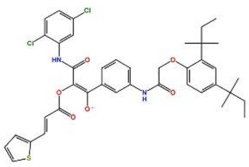<br>S:-7.5818	3257-1297<br>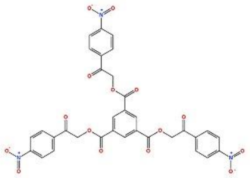<br>S:-7.5771	3773-3548<br>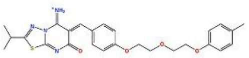<br>S:-7.5752	2368-0020<br>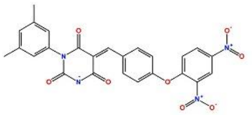<br>S:-7.5750
2036-0525<br>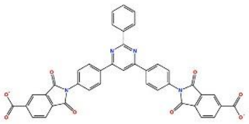<br>S:-7.5608	0940-0013<br>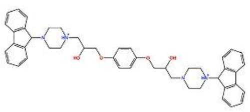<br>S:-7.5265	2036-0823<br>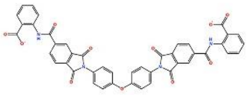<br>S:-7.5255	3202-0346<br>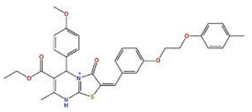<br>S:-7.5175
4013-2645<br>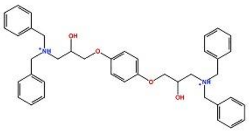<br>S:-7.5161	3448-6069<br>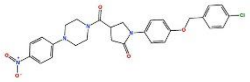<br>S:-7.5141	2324-0213<br>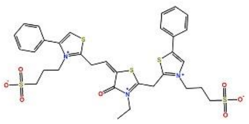<br>S:-7.5076	1477-0001<br>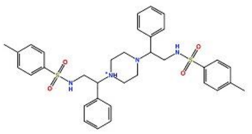<br>S:-7.5034

2030-0098 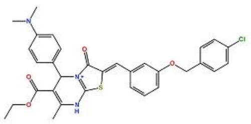 S:-7.5001	0868-0307 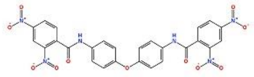 S:-7.4941	3272-1306 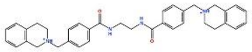 S:-7.4911	0669-0063 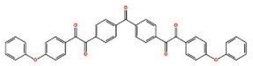 S:-7.4846
2144-0834 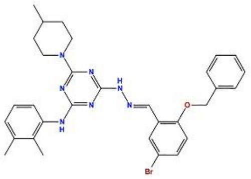 S:-7.4844	0242-0428 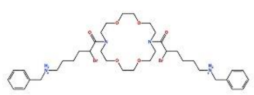 S:-7.4756	3229-0011 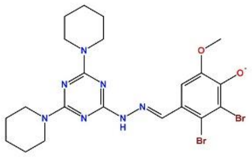 S:-7.4705	3202-0363 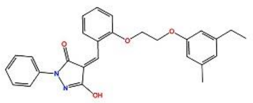 S:-7.4633
2214-0085 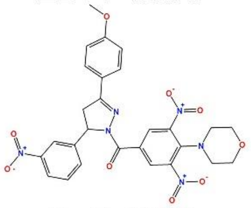 S:-7.4591	0927-0007 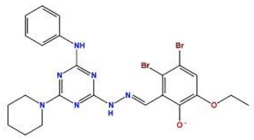 S:-7.4447	0149-0038 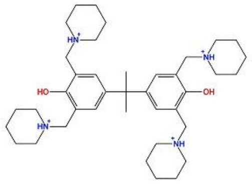 S:-7.4371	3797-0804 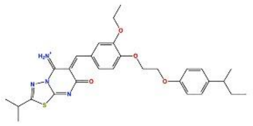 S:-7.4349
2897-1797 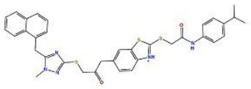 S:-7.4319	3948-1182 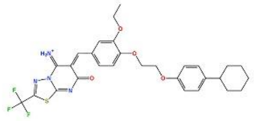 S:-7.4275	3229-0544 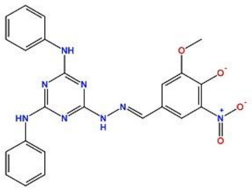 S:-7.4193	0683-0042 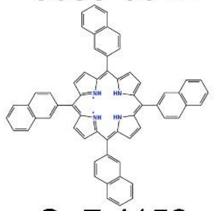 S:-7.4152
0927-0042 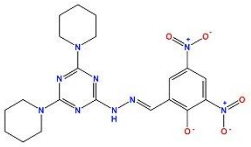 S:-7.4139	2029-0063 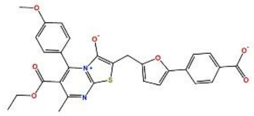 S:-7.4089	2958-7372 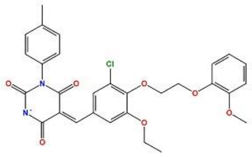 S:-7.4053	3773-4815 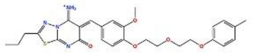 S:-7.4013

1762-0597<br>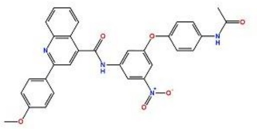<br>S:-7.3981	3773-0729<br>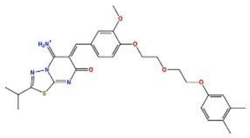<br>S:-7.3947	3184-1221<br>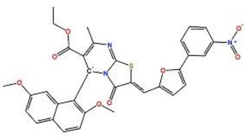<br>S:-7.3868	3706-2051<br>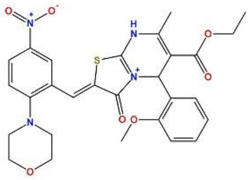<br>S:-7.3852
2641-1586<br>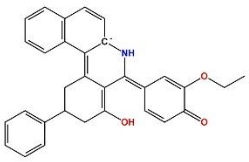<br>S:-7.3775	2897-0360<br>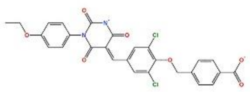<br>S:-7.3720	0102-0089<br>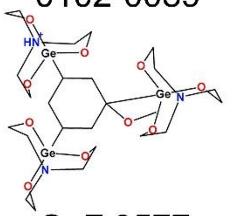<br>S:-7.3577	2425-3824<br>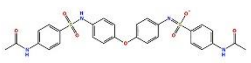<br>S:-7.3517
2958-7523<br>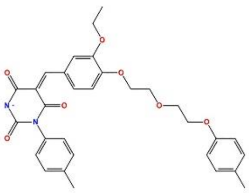<br>S:-7.3485	3235-0305<br>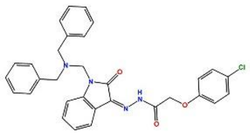<br>S:-7.3385	3797-0798<br>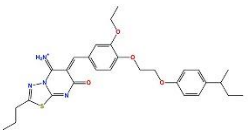<br>S:-7.3286	2030-0099<br>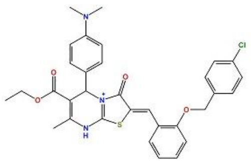<br>S:-7.3274
3948-1036<br>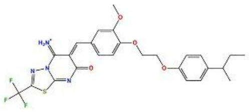<br>S:-7.3232	3389-1064<br>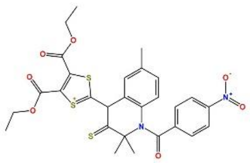<br>S:-7.3223	2958-7463<br>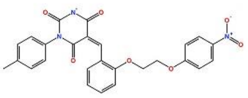<br>S:-7.3214	0878-7200<br>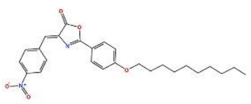<br>S:-7.3059
3537-5212<br>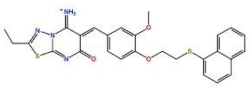<br>S:-7.2984	3948-1140<br>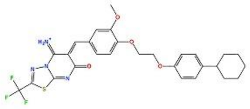<br>S:-7.2984	3797-0764<br>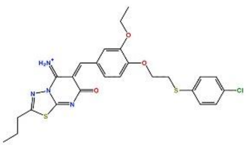<br>S:-7.2961	2368-0284<br>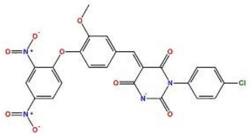<br>S:-7.2832

3537-5171 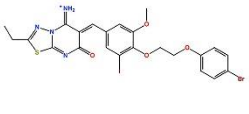 S:-7.2732	0083-0100 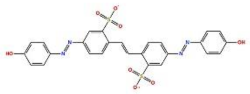 S:-7.2663	2738-1113 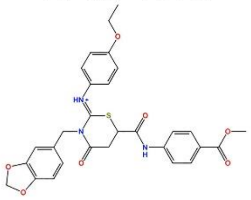 S:-7.2640	3202-0313 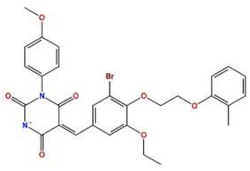 S:-7.2625
3797-0678 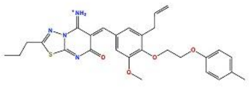 S:-7.2532	3270-0285 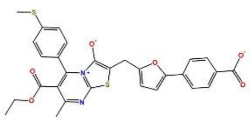 S:-7.2509	3448-3237 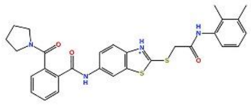 S:-7.2416	3797-0654 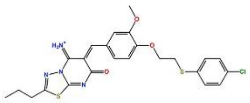 S:-7.2412
1804-0074 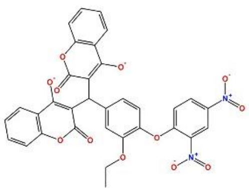 S:-7.2373	3368-0273 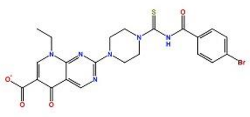 S:-7.2353	1154-0012 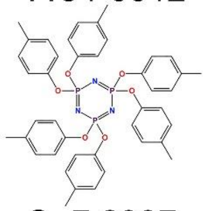 S:-7.2337	3448-2506 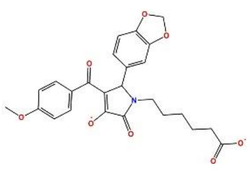 S:-7.2311
3810-0520 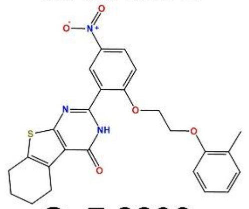 S:-7.2298	3825-7087 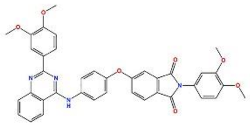 S:-7.2257	0323-0186 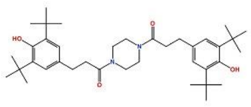 S:-7.2242	2958-7362 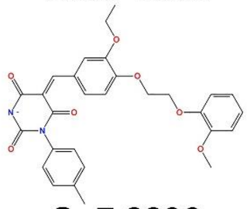 S:-7.2236
0105-0089 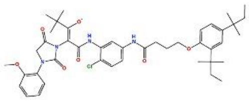 S:-7.2077	3270-0726 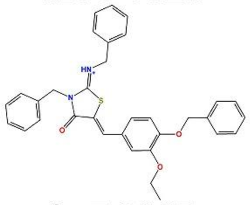 S:-7.2067	2738-0265 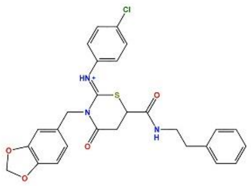 S:-7.2052	3269-0480 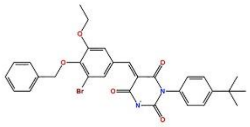 S:-7.2003

Supplementary Figure S7 Structures and docking scores of the top 100 inhibitors of the S100A9-VNN1 interaction.

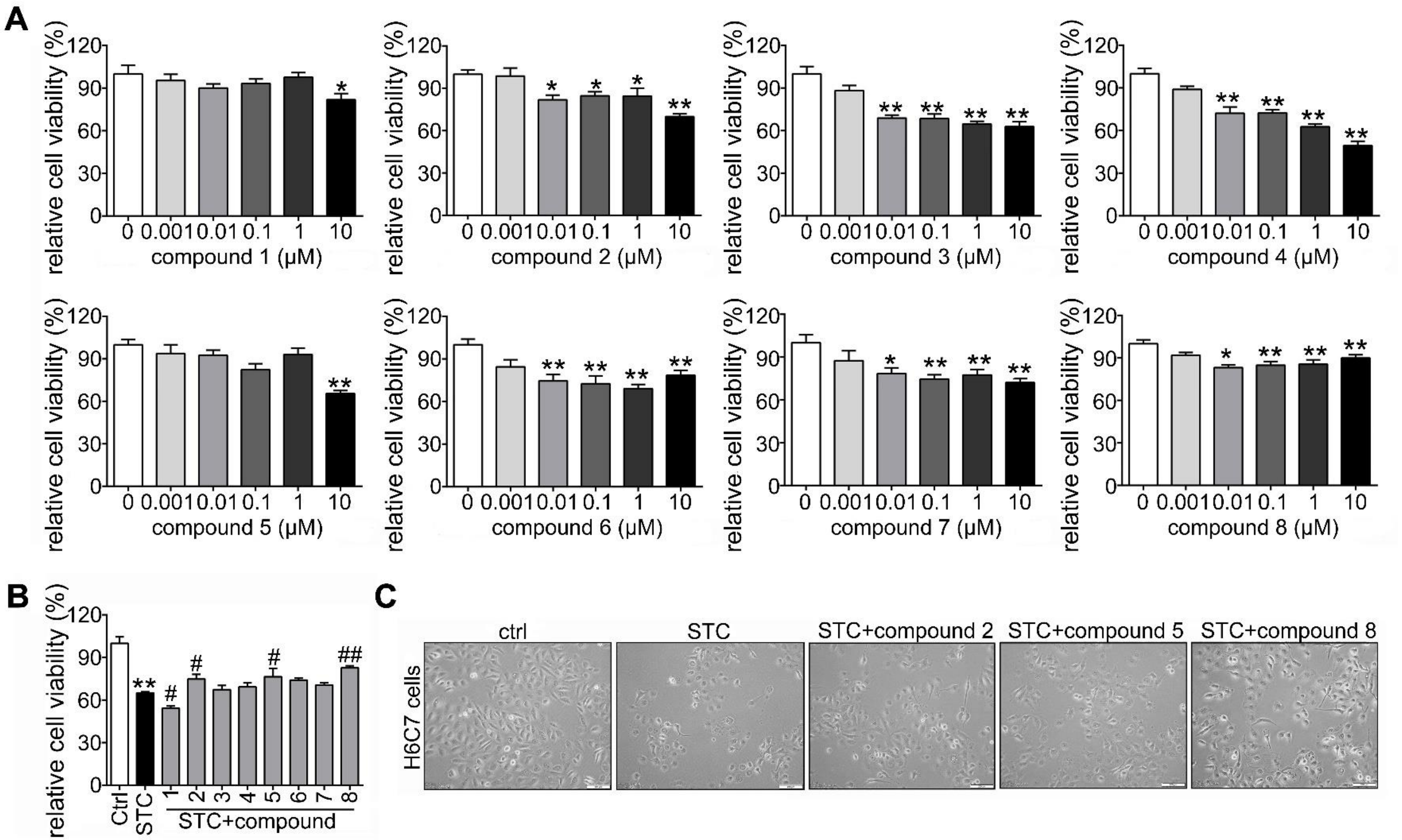

Supplementary Figure S8 (A) Toxicities of the top 8 compounds *in vitro* (n = 6). (B) Pharmacodynamics of the top 8 compounds *in vitro* (n = 6). (C) Compounds 2, 5 and 8 decreased STC-induced H6C7 cells injury. Data are presented as the mean ± SEM; *P < 0.05 and **P < 0.01 *vs.* ctrl group; $^{\#}$P < 0.05 and $^{\#\#}$P < 0.01 *vs.* STC group.

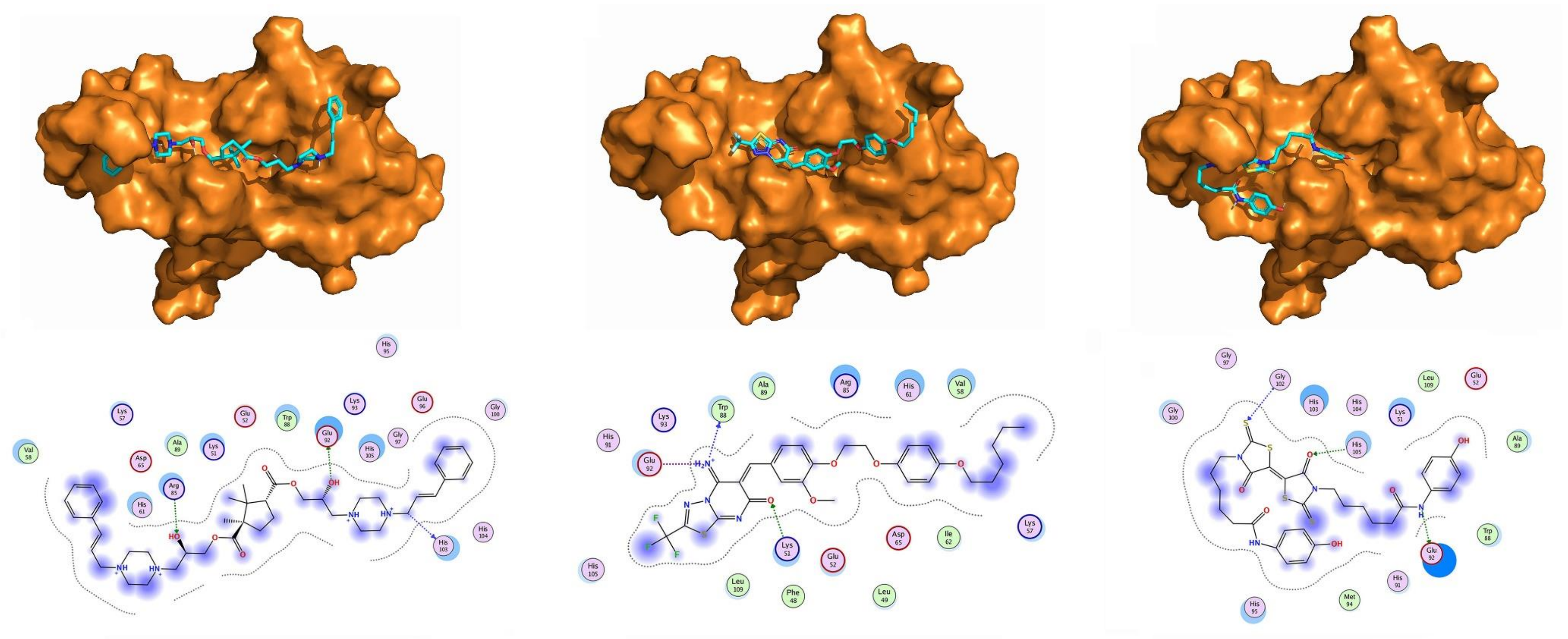

compound 2 ($C_{42}H_{60}N_4O_6$) compound 5 ($C_{28}H_{29}F_3N_4O_5S$) compound 8 ($C_{30}H_{32}N_4O_6S_4$)

Supplementary Figure S9 Binding models of $C_{42}H_{60}N_4O_6$, $C_{28}H_{29}F_3N_4O_5S$ and $C_{30}H_{32}N_4O_6S_4$ with S100A9 protein.

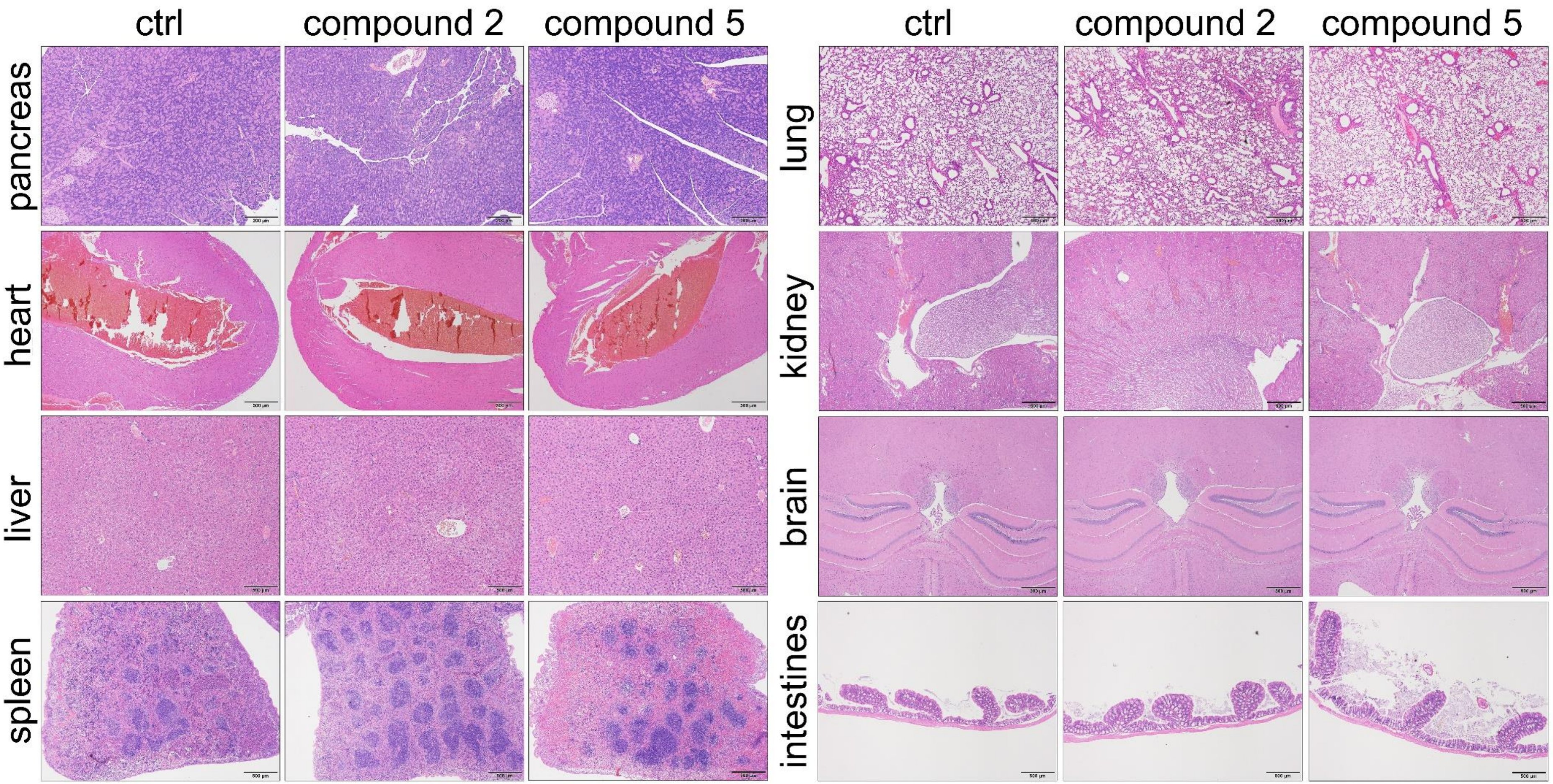

Supplementary Figure S10 HE staining results of pancreas, heart, liver, spleen, lung, kidney, brain and intestines also proved that compounds 2 and 5 have no obvious toxicities at the doses of 10 mg/kg/day for 2 days.
